# Motor domain-mediated autoinhibition dictates axonal transport by the kinesin UNC-104/KIF1A

**DOI:** 10.1371/journal.pgen.1009940

**Published:** 2021-11-29

**Authors:** Dezi Cong, Jinqi Ren, Yurong Zhou, Shuang Wang, Jingjing Liang, Mei Ding, Wei Feng

**Affiliations:** 1 State Key Laboratory of Molecular Developmental Biology, Institute of Genetics and Developmental Biology, Chinese Academy of Sciences, Beijing, China; 2 College of Life Sciences, University of Chinese Academy of Sciences, Beijing, China; 3 National Laboratory of Biomacromolecules, CAS Center for Excellence in Biomacromolecules, Institute of Biophysics, Chinese Academy of Sciences, Beijing, China; Brown University, UNITED STATES

## Abstract

The UNC-104/KIF1A motor is crucial for axonal transport of synaptic vesicles, but how the UNC-104/KIF1A motor is activated *in vivo* is not fully understood. Here, we identified point mutations located in the motor domain or the inhibitory CC1 domain, which resulted in gain-of-function alleles of *unc-104* that exhibit hyperactive axonal transport and abnormal accumulation of synaptic vesicles. In contrast to the cell body localization of wild type motor, the mutant motors accumulate on neuronal processes. Once on the neuronal process, the mutant motors display dynamic movement similarly to wild type motors. The gain-of-function mutation on the motor domain leads to an active dimeric conformation, releasing the inhibitory CC1 region from the motor domain. Genetically engineered mutations in the motor domain or CC1 of UNC-104, which disrupt the autoinhibitory interface, also led to the gain of function and hyperactivation of axonal transport. Thus, the CC1/motor domain-mediated autoinhibition is crucial for UNC-104/KIF1A-mediated axonal transport *in vivo*.

## Author summary

UNC-104/KIF1A is the founding member of the kinesin-3 family. When not transporting cargos, most kinesin-3 motors adopt an autoinhibited conformation, and how the UNC-104/KIF1A motor is activated *in vivo* is not fully understood. Here, we identified gain-of-function mutations in the motor domain or CC1 domain that significantly enhance the synaptic vesicle transport. Further biochemical and structural analyses revealed that these mutations could disrupt the CC1/motor mediated autoinhibition. Thus, our work provides a mechanistic explanation for the role of some disease-related mutations in motor hyperactivation.

## Introduction

Neurons are highly specialized for the processing and transmission of cellular signals. Most proteins necessary for synaptic terminals are transported down axon after synthesis in the cell body. UNC-104/KIF1A was originally identified through genetic screens in *C*. *elegans* and is the primary kinesin motor for anterograde axonal transport of synaptic vesicle precursors [1–3]. KIF1A-mediated axonal transport of brain-derived neurotrophic factor (BDNF) and the TrkA neurotrophin receptor is essential for hippocampal synaptogenesis and sensory neuron survival [4,5]. In addition, KIF1A also participates in controlling interkinetic nuclear migration in neural stem cells for brain development [6]. Given the aforementioned roles of KIF1A in neuronal development and synaptogenesis, it is unsurprising that mutations in the gene encoding this motor directly link with human neuronal disorders such as hereditary spastic paraparesis [7–9].

UNC-104/KIF1A belongs to the kinesin-3 subfamily [10], which can dimerize [11] and can be found as an inactive monomer or a processive homodimer [12]. The current model demonstrates that the activation of KIF1A involves the relief of its autoinhibition [13], a regulatory mechanism found in other kinesins. Autoinhibition of non-cargo-bearing kinesin motors avoids the futile consumption of ATP and prevents the potential blockage of microtubule tracks [14]. UNC-104/KIF1A contains an FHA domain sandwiched by two coiled-coils (CC1 and CC2) that together follow the neck (NC) and motor domains (MD) [15]. Previous studies showed that the CC1 and CC2 domains of KIF1A are two internal autoinhibitory segments that inhibit the motor activity. Deletions of the CC1 and CC2 domains constitutively activate the motor activity of KIF1A [16]. The CC1 domain can fold back to interact with the neck domain and inhibit the dimerization of UNC-104/KIF1A [13]. To form a dimer, this intramolecular contact between the NC and CC1 domains needs to be dissociated. The structural analysis demonstrated that the dissociated CC1 domain is required for the dimerization and activation of UNC-104/KIF1A [17,18]. In addition the CC1 domain, the intramolecular interaction between the CC2 and FHA domains is also involved in the autoinhibition of KIF1A [19]. On the other hand, the motor domain of UNC-104/KIF1A is highly conserved through the entire kinesin-3 family. In general, dimerization of kinesin-3 motors results in superprocessive motion and this property is intrinsic to the motor domain [20]. Several residues on the motor domain of kinesin-3 contribute to the enhanced processivity of this motor [21]. Moreover, gain-of-function *unc-104* mutants have been identified from genetic screens in *C*. *elegans* [22,23]. These gain-of-function mutations are mapped to various regions of UNC-104 including the motor domain [22]. Intriguingly, some mutations in the motor domain of UNC-104/KIF1A associated with human neuronal diseases can release the autoinhibited state and result in hyperactivation of the motor [22,24]. However, the mechanism underlying the involvement of the motor domain in UNC-104/KIF1A autoinhibition is not well understood.

In addition to the CC2-FHA and NC-CC1 interactions, we now revealed that the intramolecular interaction between the motor domain and the CC1 domain is also important for the autoinhibition of UNC-104/KIF1A motor. Here, the extensive genetic analysis based upon CRISPR/Cas9-mediated genome editing demonstrated that mutations disrupting the CC1/motor interactions result in hyper-activated axonal transport. Structure analysis further showed that the gain-of-function mutation on the motor region could cause an active dimeric conformation. Introducing interface-disrupting mutations into the motor domain or CC1 domain also lead to the gain of UNC-104 function in synaptic vesicle transport. In *C*. *elegans* neurons, while the wild type UNC-104 motor is distributed mainly in cell body, the gain-of-function motors tend to accumulate on particular places of neuronal processes. Once on neuronal processes, the mutant motor possesses similar motility as the wild type, suggesting the disruption of motor-domain-mediated autoinhibition results into more activated motors but not hyper-activated motors *in vivo*.

## Results

### Identification of dominant suppressors of the *unc-104* partial loss-of-function allele

**Fig 1:**
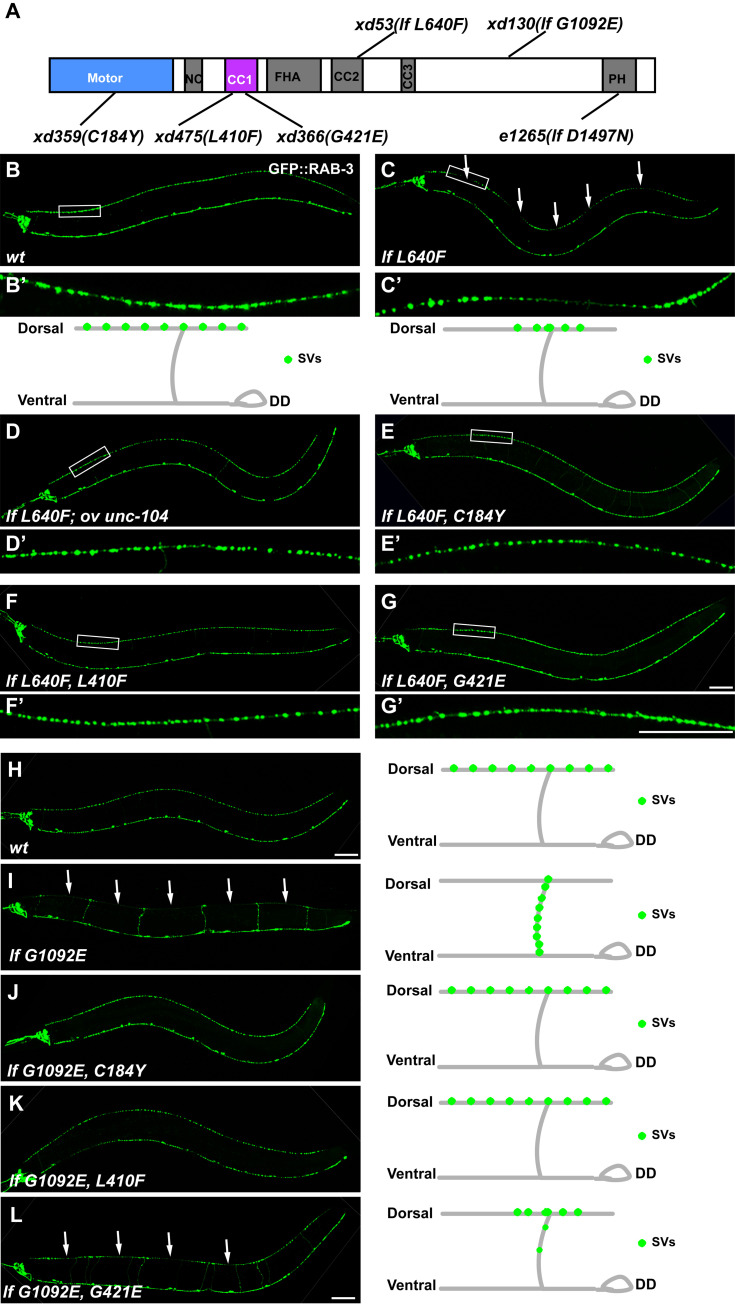
Intramolecular suppression of C184Y, L410F or G421E mutation on *unc-104(lf 640F)* and *unc-104(lf G1092E)* alleles. (A) Schematic drawing of the domain organization of UNC-104 motor protein. The mutation sites of *xd53*, *xd130*, *e1265*, *xd359*, *xd366* and *xd475* are indicated. (B-C) GFP::RAB-3 (green) driven by the P*unc-25* promoter is expressed in DD/VD neurons. (D-G) The even distribution of GFP::RAB-3 puncta on the dorsal cord is restored in *unc-104(lf L640F*, *ov unc-104) unc-104(lf L640F*, *C184Y)*, *unc-104(lfL640F*, *L410F)* and *unc-104(lf L640F*, *G421E)* animals. The boxed regions in the main images are magnified underneath. (H-L) The distribution of GFP::RAB-3 puncta in *wt* (H), *xd130(lfG1092E)* (I), *xd130 xd359 (lfG1092E*,*C184Y)* (J), *xd130 xd475 (lfG1092E*,*L410F)* (K) and *xd130 xd366 (lfG1092E*,*G421E)* (L). All images are positioned as anterior left and dorsal up. GFP::RAB-3 signals on the ventral cord are from VD neurons. White arrows indicate regions on the dorsal cord where GFP::RAB-3 puncta are absent. The schematic drawings show the synaptic vesicle distribution of DD neurons. Scale bar represents 50 μm.

In *C*. *elegans*, the cell bodies of DD motor neurons are situated on the ventral side, and they send out ventral-to-dorsal commissures to the dorsal nerve cord. After reaching the dorsal cord, each commissure splits into two branches, one extending anteriorly and the other posteriorly. These two branches form evenly distributed synaptic specializations on the dorsal muscles, which can be visualized with the synaptic vesicle marker GFP::RAB-3 driven by the *unc-25* promoter [25–27]. To identify components involved in synaptic vesicle transport, we performed a genetic screen and isolated the *xd53* mutant (Fig 1A). In wild-type animals, synaptic vesicle clusters, visualized as GFP::RAB-3 puncta, are evenly distributed along the dorsal cord (Figs 1B and S1A). In contrast, in *xd53* mutant worms, the GFP::RAB-3 signal is restricted to the region around the branching point of DD neurites at the dorsal side (Figs 1C and S1B). Through genetic mapping and genomic sequencing, we identified that the *xd53* mutation causes the change L640F in the CC2 domain of UNC-104 (Fig 1A). Expression of the wild-type *unc-104* gene efficiently rescued the abnormal distribution of GFP::RAB-3 in *xd53* animals (Fig 1D), which suggests that *xd53* is a loss-of-function allele of *unc-104*. Animals with strong loss-of-function alleles of *unc-104* have an Unc (Uncoordinated) phenotype and display synaptic vesicle accumulation in the cell body region of neuronal cells [1]. In contrast, the *xd53* animals are healthy with relatively normal locomotion. Furthermore, the synaptic vesicle clusters in *xd53* animals are found on the dorsal cord (Figs 1C, S1B and S1D), far away from the DD cell body region. Therefore, the *xd53* mutation likely causes partial functional reduction of the UNC-104 motor.

Using the *unc-104(xd53)* worms, we further performed a suppressor screen to isolate secondary site mutations which restored the synaptic vesicle distribution. From this screen, the *xd359*, *xd366*, and *xd475* mutants were isolated (Fig 1A). In *unc-104(xd53 xd359)*, *unc-104(xd53 xd366)* and *unc-104(xd53 xd475)* worms, the even distribution of GFP::RAB-3 puncta on the dorsal cord was largely reestablished, similar to the wild type (Fig 1E and 1G). In the *unc-104(xd53 xd359)/unc-104(xd53)*, *unc-104(xd53 xd366)/unc-104(xd53)* and *unc-104(xd53 xd475)/unc-104(xd53)* heterozygote animals, the GFP::RAB-3 distribution pattern resembles that in wild-type animals (S1E and S1H Fig), which suggests that *xd359*, *xd366* and *xd475* act as dominant suppressors of *xd53*. All three suppressors were difficult to separate from *xd53* through genetic recombination, indicating a tight bond to the *unc-104* locus. Through genomic DNA sequencing, we found that in addition to the *xd53(lf L640F)* mutation site, each suppressor contains a secondary mutation in the coding region of the UNC-104 protein: C184Y in *xd359*, G421E in *xd366* and L410F in *xd475* (Fig 1A).

### The non-allele suppression of *xd53* suppressors

How do the suppressors repress the mutant phenotype of *unc-104(xd53)*? One possibility is that the point mutations of *xd359(C184Y)*, *xd366(G421E)* and *xd475(L410F)* can correct the protein folding deficiency specifically caused by *xd53(lf L640F)* and therefore suppress the synaptic vesicle distribution defect only in *xd53(lf L640F)*. Alternatively, the suppressor mutations alone could lead to enhanced synaptic vesicle transport. In this case, the synaptic vesicle trafficking deficiency in other *unc-104* loss-of-function mutants may also be suppressed by these dominant suppressors. Thus, we introduced the C184Y, G421E or L410F mutation into another loss-of-function allele, *unc-104(xd130)*. The *unc-104(xd130)* allele was isolated from the same genetic screen in which *unc-104(xd53)* was identified. The mutated *unc-104* gene in *unc-104(xd130)* encodes an UNC-104 motor containing a G1092E missense mutation in the C-terminal linker region (Fig 1A). Compared to wild type (Fig 1H), the GFP::RAB-3 puncta in *xd130* are strongly reduced in the dorsal cord region (Fig 1I). Meanwhile, the GFP signal appears on the commissure region of DD neurons (Fig 1I). In addition, the *unc-104(xd130)* animals display an uncoordinated (Unc) locomotion phenotype (S2A and S2E Fig). Using the CRISPR/Cas9 technique [28], the C184Y or L410F mutation was introduced into *xd130(lf G1092E)*, which restored the even distribution of GFP::RAB-3 puncta on the dorsal cord (Fig 1J and 1K). Introducing the G421E mutation into *xd130(lf G1092E)* resulted in a redistribution of the synaptic vesicle clusters to the dorsal cord region. We noticed, however, that the GFP::RAB-3 puncta in *unc-104(lf G1092E*, *G421E)* were not evenly spread along the whole dorsal cord but instead tended to localize around the dorsal branching point of DD neurons (Fig 1L). Hence, compared to C184Y or L410F, the G421E mutation has a weaker suppression effect on *xd130(lf G1092E)*. We further examined the Unc phenotype and found that the *unc-104(xd130)* locomotion defect was considerably suppressed by the C184Y, L410F or G421E suppressor mutation (S2B–S2E Fig). *e1265* is a strong loss-of-function allele of *unc-104*. The mutated *unc-104* gene in *unc-104(e1265)* encodes an UNC-104 motor containing a D1497N mutation in the C-terminal PH domain, which may reduce the interaction with cargo [23]. In *e1265* animals, the synaptic vesicles are retained at the cell bodies [23] (S3 Fig). Using CRISPR/Cas9-based genome editing, we successfully generated *unc-104(lf D1497N*, *C184Y)*, *unc-104 (lf D1497N*, *G421E)*, and *unc-104(lf D1497N*, *L410F)* worms. Intriguingly, the abnormal synaptic vesicle accumulation defect of *e1265(lf D1497N)* could not be suppressed by *xd359(C184Y)*, *xd366(G421E)* or *xd475(L410F)* (S3 Fig). Based upon above observations, we suspected that the *xd359(C184Y)*, *xd366(G421E)*, and *xd475(L410F)* suppressors may specifically affect the non-cargo-binding aspect of UNC-104 motors. Alternatively, fewer activated motors *in vivo* might lead to fewer motors binding to cargo and motors not binding to cargo may undergo degradation [23]. In this situation, the suppression of *xd359(C184Y)*, *xd366(G421E)* or *xd475(L410F)* on *xd53* or *xd130* might be due to more active motors which lead to more motors being recruited on the cargo.

### Abnormal accumulation of synaptic vesicles in suppressor mutant animals

How then do the C184Y, L410F and G421E mutations lead to suppression on both *xd53(lf L640F)* and *xd130(lf G1092E)*? To assess the transport function of the UNC-104^C184Y^, UNC-104^L410F^ and UNC-104^G421E^ motors *in vivo*, we created *xd359(C184Y)*, *xd475(L410F)* and *xd366(G421E)* mutant animals utilizing the CRISPR/Cas9 technique. The *xd359(C184Y)*, *xd475(L410F)* and *xd366(G421E)* animals are healthy with normal locomotion. In DD motor neurons, we found that the synaptic vesicle clusters were evenly distributed along the dorsal cord in *xd359(C184Y)*, *xd366(G421E)* and *xd475(L410F)* mutant animals (S4A–S4D Fig), similar to wild type. The finding above suggests that axonal transport is generally preserved for the UNC-104^C184Y^, UNC-104^L410F^ and UNC-104^G421E^ motors.

**Fig 2:**
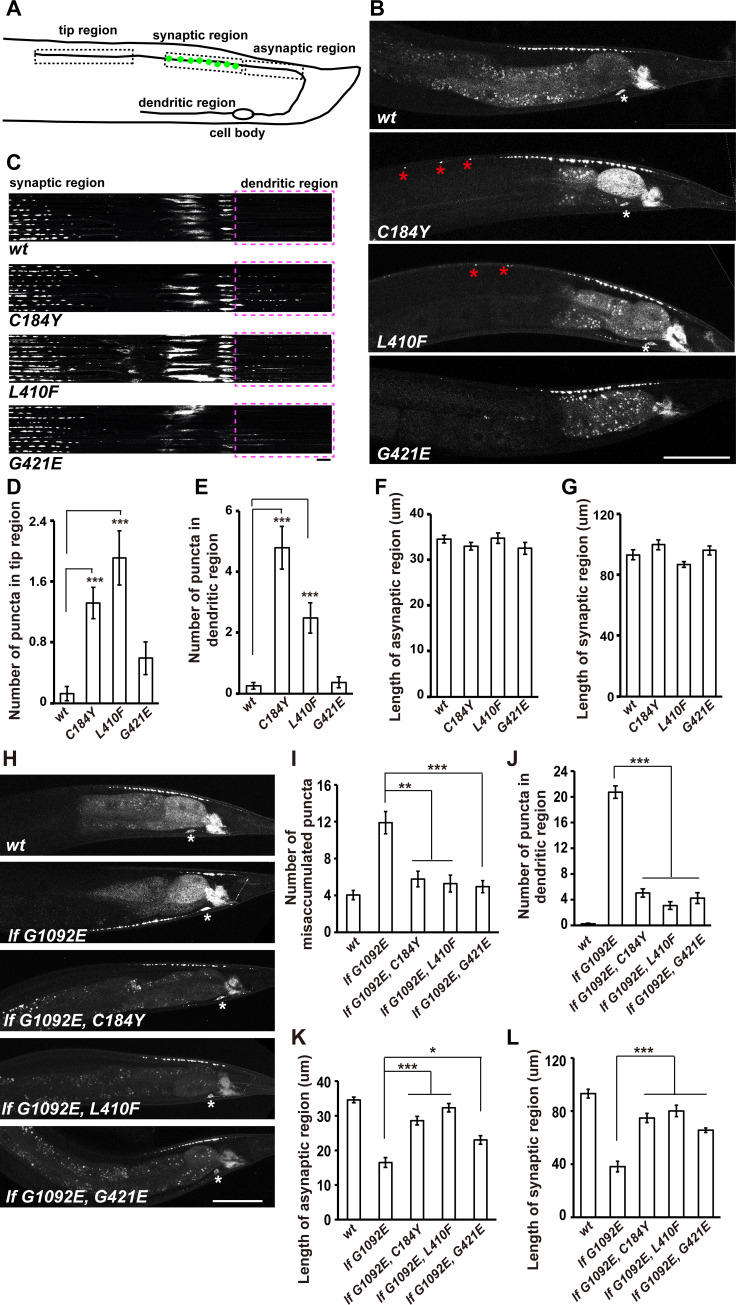
Abnormal synaptic vesicle accumulation in suppressor mutants. (A) Schematic drawing of the DA9 neuron. Different regions of DA9 are boxed and labeled. The synaptic vesicle clusters (green) are indicated in the synaptic region. (B) Puncta formed by GFP::RAB-3 under the control of P*itr-1* appear in the tip region in *unc-104(C184Y)* and *unc-104(L410F)* animals (asterisks). Scale bar represents 50 μm. (C) GFP::RAB-3 signals appear in the dendritic region in *unc-104(C184Y)* and *unc-104(L410F)* animals. Line scan images of DA9 neurons. Ten DA9 neurons from independent animals were scanned and aligned. Scale bar represents 5 μm. Dashed boxes indicate the dendritic region. (D and E) Numbers of GFP::RAB-3 puncta in the tip region and the dendritic region. (F and G) Quantification of the length of the asynaptic region and the synaptic region. (H-L) The abnormal synaptic accumulation defect in *unc-104(lf G1092E)* could be suppressed by C184Y, L410F, or G421E mutation on UNC-104. (I) Quantification of the GFP::RAB-3 puncta in the asynaptic region and commissure region. (J) Quantification of the GFP::RAB-3 puncta in the dendritic region. (K and L) Quantification of the length of the asynaptic region and the synaptic region. *P<0.05, **P<0.01, ***P<0.001, one-way ANOVA with Tamhane’s T2 test. Mean ± SEM, N> = 19 worms for each genotype.

The six DD neurons extend their dorsal neurites to overlap each other [29] and therefore the synaptic vesicle distribution in individual DD neurons cannot be revealed by the P*unc-25*::GFP::RAB-3 marker, which highlights synaptic vesicle clusters in all DD neurons. Hence, we turned to the DA9 neuron. Using the P*itr-1*::GFP::RAB-3 reporter, the synaptic vesicle clusters of DA9 neurons can be visualized in a specific region along the dorsal axon, leaving the other regions (the dorsal distal tip region, dorsal proximity, the commissure and the ventral dendritic region) devoid of pre-synaptic structures [30] (Fig 2A). Interestingly, when we examined the *unc-104(C184Y)* and *unc-104(L410F)* mutant animals, we found that in addition to the synaptic region, ectopic GFP::RAB-3 puncta appeared in the dorsal distal tip region (Fig 2B and 2D) and the ventral dendritic region (Fig 2C and 2E), which is consistent with the role of constitutive UNC-104 motors *in vivo* [22,24]. The *unc-104(G421E)* mutant animals did not display apparent ectopic synaptic vesicles at the distal region of either the dorsal or ventral process. We suspected that the G421E mutation has a relatively weak gain-of-function effect on UNC-104. Indeed, while the synaptic vesicle distribution in *xd53(lf L640F)* or *xd130(lf G1092E)* was restored or partially restored by G421E, the G421E mutation alone did not significantly augment the axonal transport in DA9 neurons. The length of the synaptic and asynaptic region is not altered by *unc-104(C184Y)*, *unc-104(L410F)* or *unc-104(G421E)* (Fig 2F and 2G), which suggests that the suppressor mutations do not affect synapse formation in general.

In *xd53(lf L640F)* mutants, synaptic vesicles abnormally accumulate in the commissure area of DA9 (S4E and S4F Fig), and either C184Y or G421E mutation could suppress this phenotype (S4G–S4I Fig). The *xd130(lf G1092E)* mutants also display ectopic synaptic vesicles on the commissure area and this phenotype could be suppressed by C184Y, L410F or G421E mutation (Fig 2H and 2I). In addition, synaptic vesicle clusters appear in the dendrite region in *xd130(lf G1092E)*. Based on the average number of GFP::RAB-3 puncta, the *xd130(lf G1092E)* display a much stronger dendritic accumulation defect than *unc-104(C184Y)* or *unc-104(L410F)*. Introducing the C184Y, L410F or G421E mutation could significantly reduce the synaptic vesicle clusters in the dendrite region in *xd130(lf G1092E)* (Fig 2H and 2J). Furthermore, the synaptic vesicle clusters at the synaptic region could also be restored by the C184Y, L410F or G421E mutation (Fig 2H and 2L).

### Increased cargo movement in the suppressor mutants

**Fig 3:**
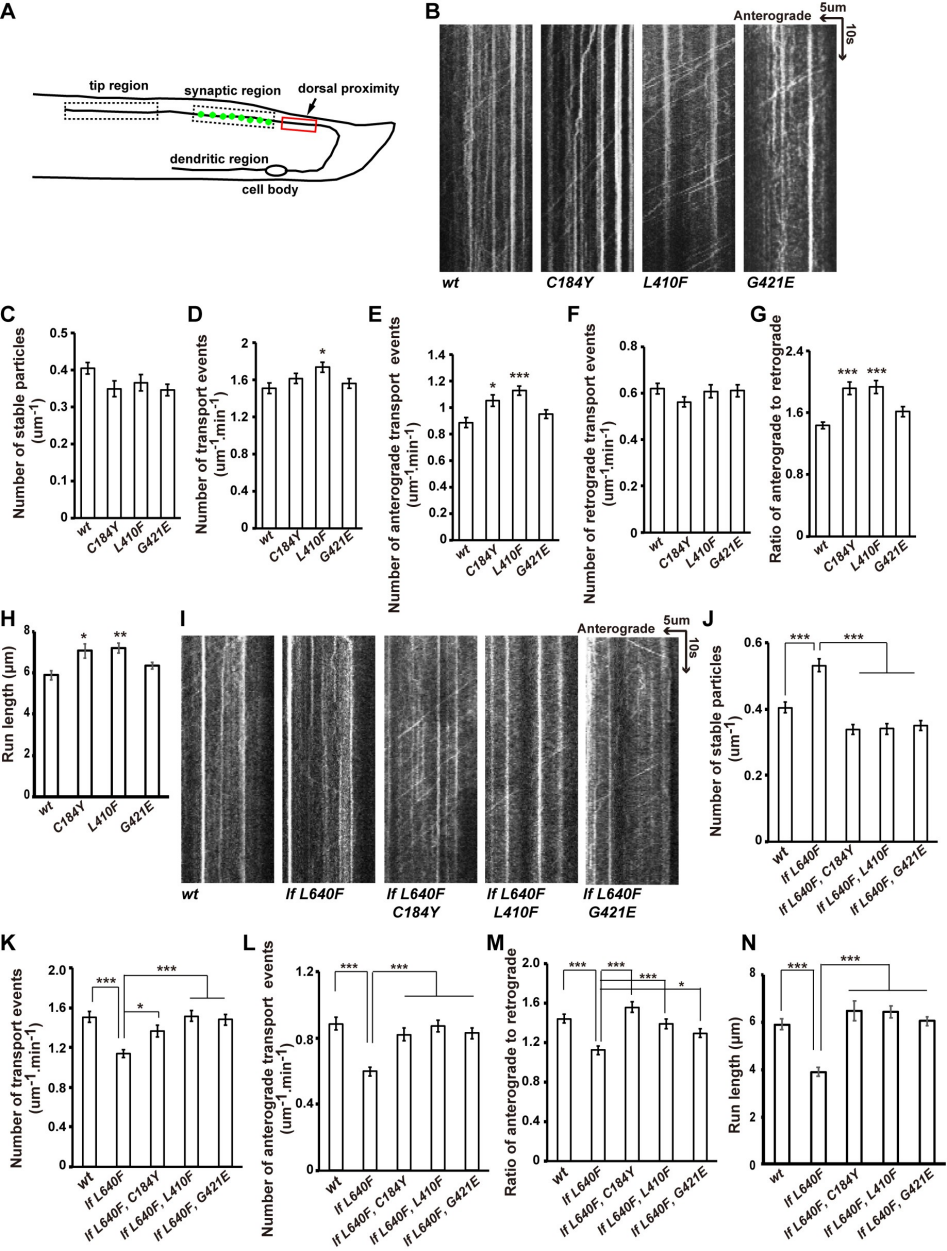
Synaptic vesicle transport is more active in suppressor mutants. (A) Schematic drawing of the wild-type DA9 neuron. Kymograph analysis was performed in the dorsal proximity region. (B) Representative kymograph images showing transport events in wild type (*wt*), *unc-104(C184Y)*, *unc-104(L410F)* and *unc-104(G421E)*. Time and length are on the y axis and x axis, respectively. (C) The number of stable GFP::RAB-3 particles in a 1-μm section within 1 min. (D) The total number of transport events (anterograde plus retrograde) was measured by counting the number of GFP::RAB-3 particles that passed through this 1-μm zone during the 1-min recording time.* P<0.05, one-way ANOVA with Tukey’s test. (E and F) The number of anterograde (E) or retrograde (F) transport events was measured by counting the number of GFP::RAB-3 particles that passed through the 1-μm zone in each direction during the 1-min recording time. * P<0.05, ***P<0.001, one-way ANOVA with Tukey’s test. (G) The ratio of anterograde transport events to retrograde transport events. (H) The run length of GFP::RAB-3 particles is increased in *unc-104(C184Y)* and *unc-104(L410F)* mutants.***P<0.001, one-way ANOVA with Tamhane’s T2 test. (I-N) The dynamic movement of GFP::RAB-3 particles in *unc-104(lf L640F)* could be restored by C184Y, L410F or G421E mutation. * P<0.05, **P<0.01, ***P<0.001, one-way ANOVA test. Mean ± SEM, N = 30 worms for each genotype.

We further measured the axonal transport by performing kymograph analysis in the dorsal proximity region, through which the synaptic vesicles are delivered to their final destination, the synaptic region (Fig 3A and 3B). Parameters associated with GFP::RAB-3 moving particles, particularly anterograde movements, are significantly increased for UNC-104^C184Y^ and UNC-104^L410F^ compared to wild-type UNC-104 (Fig 3C and 3G). In addition, the cargo segment run length is increased for UNC-104^C184Y^ and UNC-104^L410F^ (Fig 3H). In contrast, the number of stable GFP::RAB-3 puncta and the number of retrograde movements in the presence of UNC-104^C184Y^ or UNC-104^L410F^ are indistinguishable from wild-type UNC-104 (Fig 3C and 3F).

We also examined the cargo movement in *xd53(lf L640F)*. Compared to the wild type, the anterior movement and the cargo segment run length significantly decreased for UNC-104 ^lf L640F^ (Fig 3L and 3N). Meanwhile, the presence of the C184Y, L410F or G421E mutation could efficiently suppress the decreased anterior movement and cargo segment run length caused by UNC-104 ^lf L640F^ (Fig 3I and 3N).

### The UNC-104^C184Y^ motors are absent from cell bodies and accumulate on neuronal processes

**Fig 4:**
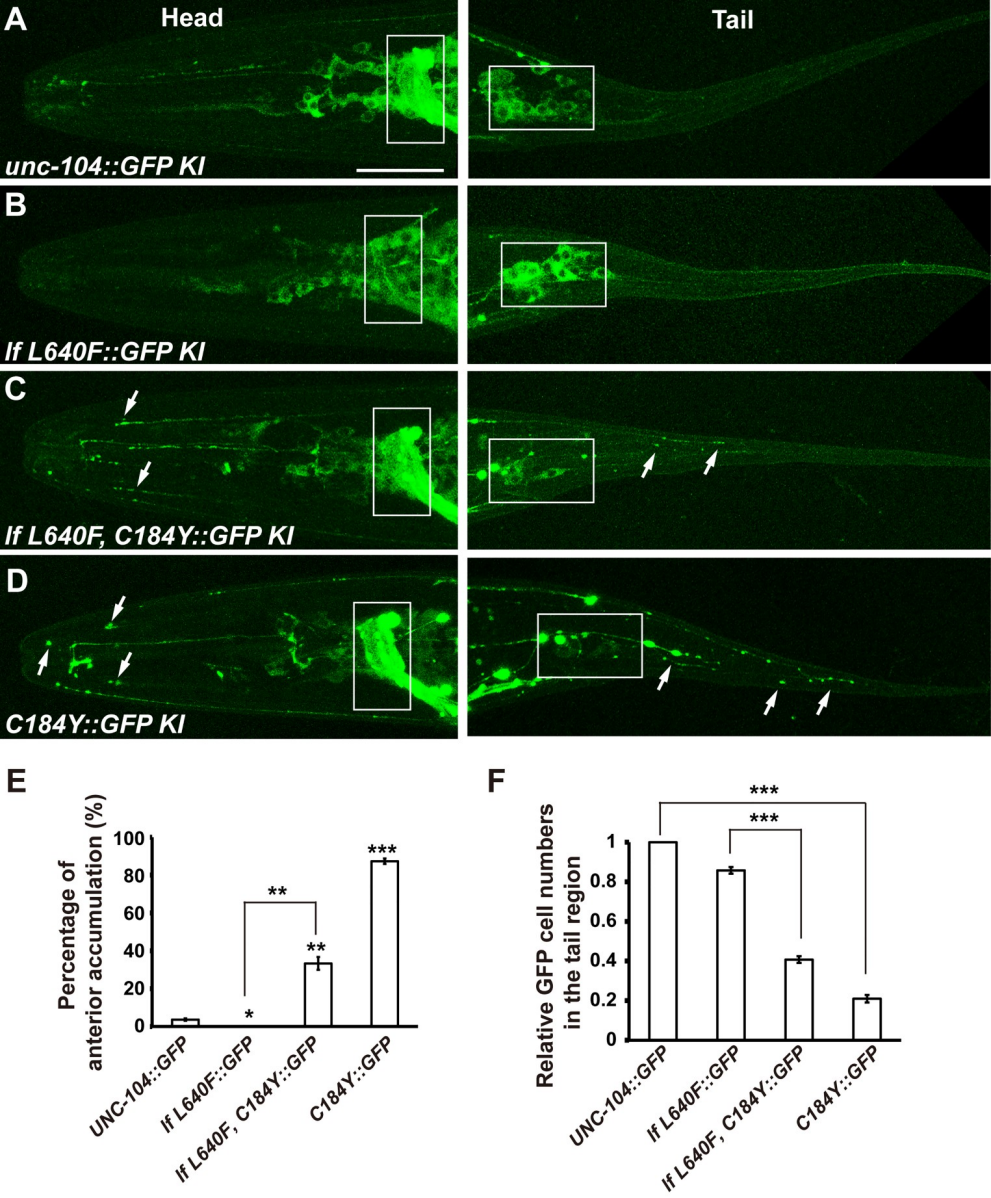
Endogenous localization of wild-type and mutant UNC-104motors. (A and B) The wild-type and UNC-104^lf L640F^motor (GFP KI, green) are diffusely distributed in neuronal cell bodies and processes. (C and D) The UNC-104^lf 640F, C184Y^and UNC-104^C184Y^ motor (GFP KI, green) tend to accumulate on neuronal processes and are generally absent from neuronal cell bodies. Boxes indicate part of the ring ganglion (left) or pre-anal ganglion (right). White arrows indicate some of the neuronal termini. Scale bar represents 25 μm. (E) Quantification of the percentage of animals showing anterior accumulation of GFP signal. *P<0.05, ** P<0.01, *** P<0.001, one-way ANOVA with Tamhane’s T2 test. 300 worms were observed for each genotype. Mean ± SEM, N = 6. (F) Quantification of the GFP positive cells in the tail region. *** P<0.001, one-way ANOVA with Tamhane’s T2 test. Mean ± SEM, N = 30.

Next, we examined the endogenous protein distribution of the wild type and mutant UNC-104 motors *in vivo*. UNC-104^C184Y^ likely possesses the most robust synaptic vesicle transport activity (Figs 2 and 3). Hence, we created GFP knock-in lines for UNC-104 and UNC-104^C184Y^ using the CRISPR/Cas9 technique. We found that the UNC-104^C184Y^ mutant motor protein expression level in worms is similar to that of wild-type UNC-104 (S5A and S5B Fig), which suggests that the stability of UNC-104 protein is not affected by the C184Y mutation. However, the wild-type UNC-104 motor was generally diffusely distributed in the neuronal cell bodies and neuronal processes (Fig 4A). In contrast, the UNC-104^C184Y^::GFP forms bright dots on neuronal processes (Fig 4D) and the fluorescence signal from UNC-104^C184Y^::GFP was much reduced in the cell body region compared to wild type UNC-104::GFP (Fig 4A and 4D).

We further created the UNC-104^lf L640F^::GFP knock-in line and found that the UNC-104^lf L640F^::GFP was diffusely distributed in the neuronal cell bodies, which is more or less resembles wild type UNC-104::GFP. We noticed that the UNC-104^lf L640F^::GFP is lower than wild type UNC-104::GFP in the nerve ring region (Fig 4A and 4B), suggesting the UNC-104^lf L640F^ motors on neuronal processes maybe reduced. The C184Y mutation could largely restore the GFP signal on the nerve ring region for UNC-104^lf L640F^::GFP (Fig 4C). In addition, the UNC-104^lf L640F C184Y^::GFP accumulates on neuronal processes, and the fluorescence signal from UNC-104^lf L640F C184Y^::GFP is greatly reduced from cell body region (Fig 4E and 4F). In general, the UNC-104^lf L640F C184Y^::GFP distribution pattern is similar to UNC-104^C184Y^::GFP, implying that the UNC-104(lf L640F) function could be restored or even strengthened by C184Y mutation.

**Fig 5:**
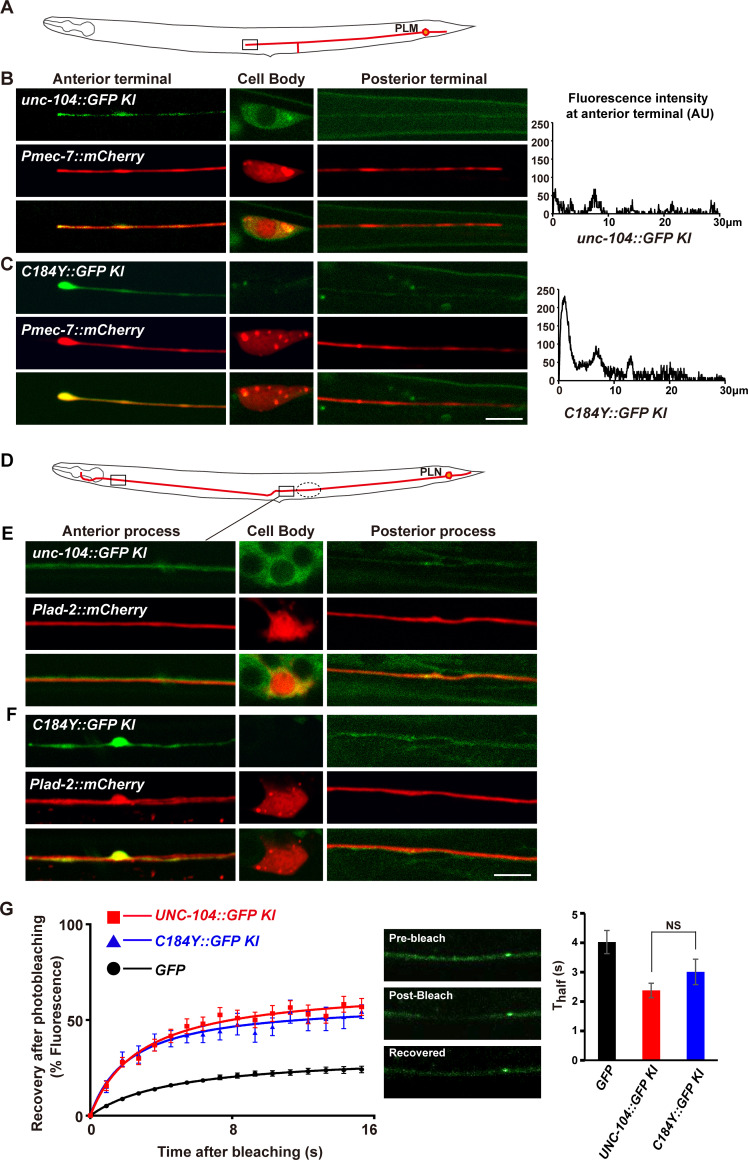
The UNC-104^C184Y^ motors accumulate on neuronal processes of PLM and PLN neurons. (A) The schematic drawing illustrates the PLM neuron (red) in *C*. *elegans*. Boxed region indicates the terminal region of the anterior process of PLM. (B) The UNC-104::GFP distribution in PLM neuron. The PLM neuron is in red. The UNC-104::GFP is in green. (C) The UNC-104^C184Y^::GFP distribution in PLM neuron. The PLM neuron is in red. The UNC-104^C184Y^::GFP is in green. Scale bar represents 5 μm. (D) The schematic drawing illustrates the PLN neuron (red) in *C*. *elegans*. (E) The UNC-104::GFP (green) is diffusely distributed in PLN neuron (red). (F) The UNC-104^184Y^::GFP (green) is accumulated on the neuronal process of PLN neuron (red), particularly in the two regions indicated by solid boxes. Scale bar represents 5 μm. (G) The FRAP analysis was conducted in the region encircled by dashed line. Curves of fluorescence after photobleaching of wild type UNC-104::GFP and UNC-104^C184Y^::GFP on PLN neuron. *N* = 28 for wild type UNC-104::GFP and *N* = 24 for UNC-104 ^C184Y^::GFP.

To reveal the specific region on which the UNC-104^C184Y^ mutant motor is accumulated, we introduced the UNC-104^C184Y^::GFP into the neuronal marker, specifically labelling PLM neuron [31]. PLM neuron has its cell body located in the tail region and sends out an anterior and a posterior process along the lateral side of the worm body (Fig 5A). On the lateral side, the PLM neuron is well separated from other neurons and the neuronal processes of PLM could be clearly identified. Before reaching the vulva region, the PLM anterior neurite branches out two processes: one projects continuously forward and the other goes ventrally and forms *en passant* chemical synapses with neurons in the ventral nerve cord. We found that the UNC-104^C184Y^::GFP generally lacked in the PLM cell body, compared to wild type UNC-104::GFP (Fig 5B and 5C). Instead, it is specifically accumulated in the terminal region of the anterior process of PLM (Figs 5C and S5C). The neuronal morphology of PLM is not altered by UNC-104^C184Y^::GFP (S5D Fig). In addition, the synaptic vesicles are still localized in the synaptic area of PLM in UNC-104^C184Y^::GFP worms, similarly to wild types (S5E Fig). Thus, neuronal development is not affected by C184Y mutation in general.

Does UNC-104^C184Y^ motor accumulate only at the terminal region of neuronal process? To address this question, we further examined PLN neuron. The PLN neuron is situated in the tail region and sends out an anterior and a posterior process along the lateral side of the worm (Fig 5D). By examining the transgenic worms in which the PLN neuron is specifically visualized, we found that the UNC-104^C184Y^::GFP was accumulated on two places of the anterior process of PLN (solid boxes in Fig 5D and 5F). The end of the PLN anterior process is buried in nerve region in which many neuronal processes fasciculate together. Therefore, it is difficult to identify UNC-104^C184Y^::GFP signal specifically coming from PLN terminal in this region. Nevertheless, above observations suggest that in addition to the neuronal terminals, UNC-104^C184Y^ motors could form accumulates on neuronal processes as well.

We further examined the dynamic movement of wild type and UNC-104^C184Y^ mutant motors *in vivo*. By applying fluorescence recovery after photobleaching (FRAP) on the anterior process of PLN neuron (encircled in dashed line in Fig 5D), we found that the photo recovery process of UNC-104^C184Y^::GFP resembled wild type (Fig 5G), suggesting that the dynamic movement of UNC-104^C184Y^ is similar to wild type motor on the neuronal process *in vivo*.

### Disruption of CC1/motor domain-mediated autoinhibition by the dominant suppressors

**Fig 6:**
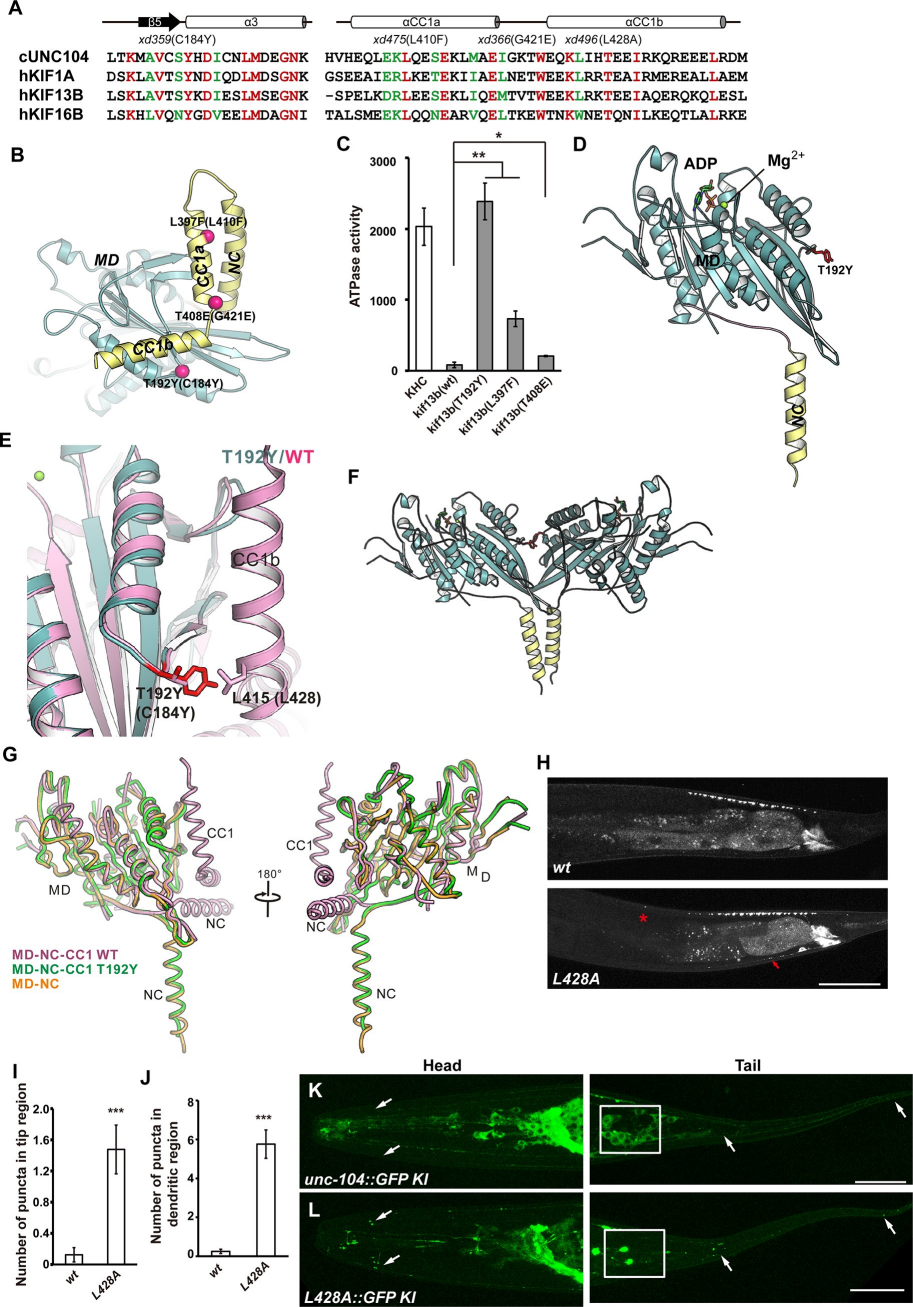
Conservation of the MD residues associated with autoinhibition. (A) Structure-based sequence alignment of the binding regions between MD and NC-CC1. Identical residues are colored in red, and highly conserved residues in green. The secondary structures and residue numbers of UNC-104 are marked at the top. The mutation sites of *xd359*, *xd475*, *xd366* and *xd496* are also indicated. αCC1a and αCC1b are the two helixes within CC1. (B) Mapping of the mutation sites on the structure of KIF13B MD-NC-CC1. (C) Microtubule-stimulated ATPase activity of the KIF13B MD-NC-CC1 fragment containing the T192Y, L397F or T408E mutation. Bars represent Mean ± SD. n = 4 independent experiments, * P<0.05, one-way ANOVA with Tamhane’s T2 test. (D) A ribbon diagram of the structure of the T192Y-MD-NC-CC1 subunit. The motor domain, NL, NC and CC1 are colored in pale cyan, pink, yellow and light orange, respectively. The sidechain of T192Y is shown and highlighted in red. (E) Structural comparison of the T192Y-MD-NC-CC1 and MD-NC regions. The structure of the T192Y-MD-NC-CC1mutant dimer can be superimposed well on the active MD-NC dimer. (F) A ribbon diagram of the structure of the T192Y-MD-NC-CC1 dimer. (G) Structure-based comparison between the wild type (WT) MD-NC-CC1 and the mutant T192Y-MD-NC-CC1. (H) Puncta formed by GFP::RAB-3 under the control of P*itr-1* appear in the tip region in *unc-104(L428A)* animals (asterisks). Scale bar represents 50 μm. (I and J) Numbers of GFP::RAB-3 puncta in the tip region (I) and the dendritic region (J).***P<0.001, unpaired student t-test. Mean ± SEM, N> = 20. (K and L) Compared to wild type UNC-104::GFP KI (green), the UNC-104^L428A^::GFP is accumulated on neuronal processes and absent from the cell body region. Boxes indicate part of the ring ganglion (left) or pre-anal ganglion (right). White arrows indicate some of the neuronal termini. Scale bar represents 25 μm.

Previous studies showed that the motor domain of kinesin-3 family member KIF13B could interact with both NC and CC1 to form a compact self-folded monomer for autoinhibition [32]. KIF13B is highly expressed in the liver and localized on the sinusoidal plasma member of hepatocytes [33]. KIF13B binds to centaurin-α1 and hDLG1 via its FHA and MBS (membrane-associated guanylate kinase-homologue binding stalk) domains [34–36]. The cargo of KIF13B has not yet been clearly identified and whether the motor-mediated autoinhibition of KIF13B could be applied to UNC-104/KIF1A has not been revealed. Intriguingly, when we mapped the C184Y, L410F and G421E point mutations onto the structure of the autoinhibited MD-NC-CC1 monomer of KIF13B (Fig 6A), we found that all three mutations are located in inter-domain interfaces within the MD-NC-CC1 monomer. Specifically, the three residues C184, L410 and G421 in UNC-104 (corresponding to T192, L397 and T408 in KIF13B) are located in the MD-CC1b interface, the NC-CC1a interface and the CC1a-CC1b turn, respectively (Fig 6B). Mutations of C184 and L410 are predicted to directly disrupt the inter-domain packing for autoinhibition, while mutation of G421 is predicted to impact the sharp turn between CC1a and CC1b, which may also somewhat interfere with the packing between CC1 and the other two domains.

Based on the structural analysis, we tried to make these three point mutations in the MD-NC-CC1 fragment of UNC-104 and evaluate their potential effects on the microtubule-stimulated ATPase activity *in vitro*. Firstly, we made the MD-NC-CC1 fragment of KIF13B, which could be purified in sufficient quantity and quality for structural and biochemical characterization [32]. We introduced the three point mutations (T192Y, L397F and T408E) individually into the MD-NC-CC1 fragment of KIF13B and purified the mutant proteins after expression in bacteria. As expected, in comparison to the autoinhibited wild type, both the T192Y and L397F mutations significantly restored the ATPase activity of the MD-NC-CC1 fragment, while the T408E mutation only moderately restored it (Fig 6C). This suggests that these mutations can indeed disrupt the CC1/motor domain-mediated autoinhibition. Accordingly, the T192Y and L397F mutations also resulted in conformational changes of the MD-NC-CC1 fragment, as indicated by the obvious shifts of the elution volume of these two mutants in analytical gel-filtration analysis (S6A Fig). These biochemical data are consistent with the hyperactive properties of the gain-of-function suppressor alleles of *unc-104* containing the corresponding point mutations (Figs 1–3). Notably, in agreement with the more positive effects of the T192Y and L397F mutations on restoring the ATPase activity of the KIF13B MD-NC-CC1 fragment (Fig 6C), the C184Y and L410F mutations in UNC-104 also seem to have more impact on the motor hyperactivity than the G421E mutation (Figs 1–3). Secondly, we purified UNC-104 (MD-NC-CC1, residue 1–446) using a bacterial expression system following the protocol from a previous report [11] and further performed the MT-stimulated ATPase assay. Different from KIF13B, the MD-NC-CC1 fragment of UNC-104 shows the moderate MT-stimulated ATPase activity, probably due to that the distinctive long hinge between the NC and CC1 domains may somewhat promote the truncated UNC-104 motor into an active state and the motor may exist in an equilibrium of the inactive-to-active states [13,19,37]. Further point mutations (C184Y, L410F and G421E) were made on this truncated UNC-104 motor. As expected, the C184Y mutant undergo a significant enhancement in its ATPase activity, other mutants also show comparable or slightly increased ATPase activity compared to the wild-type protein (S6B Fig), suggesting that these mutations disrupt the equilibrium and drive the motor into an active state.

Previous studies showed that gain-of-function alleles of *unc-104* could suppress the synaptic vesicle mis-localization defect of *arl-8*. We found that the synaptic vesicle transport defect in *arl-8* indeed could be suppressed by *xd359(C184Y)*, *xd475(L410F)* or *xd366(G421E)* (S7 Fig), further supporting the notion that C184Y, L410F and G421E mutations lead to gain-of-function of *unc-104*.

### Structure of the T192Y-MD-NC-CC1 dimer of KIF13B

Because we could not obtain sufficient quantity and quality of UNC-104 protein for structural characterization, to further investigate the mechanism underlying the mutation-induced activation, we performed crystal screening of the three mutants of the KIF13B MD-NC-CC1 fragment. We successfully crystallized the T192Y-MD-NC-CC1 mutant and determined its structure by the molecular replacement method. The structure was refined to 2.3 Å with Rwork/Rfree of 18.2%/24.1% (Fig 6D–6G and S1 Table). In the structure of the T192Y-MD-NC-CC1 mutant, we could trace the electron densities of the motor domain (with bound ADP-Mg^2+^) and NC, but not of CC1, possibly due to the intrinsic flexibility of CC1 upon dissociation from the motor domain (S6C Fig). Although only one molecule was found in the asymmetric unit of the crystal, a MD-NC dimer could be formed by a crystallographic two-fold symmetry axis (Fig 6F). This demonstrates that the T192Y-MD-NC-CC1 mutant can indeed adopt an active dimeric conformation, mediated by the NC coiled-coil. Upon superimposition, the structure of the T192Y-MD-NC-CC1 mutant was very similar to that of the active MD-NC dimer (Fig 6G), which also supports the notion that this is the active conformation of the T192Y-MD-NC-CC1 mutant. To reveal the mechanism of T192Y-mediated activation, we further compared the structure of the T192Y-MD-NC-CC1 mutant with that of the wild type. In the autoinhibited conformation of the wild type MD-NC-CC1, T192 sits in the interaction interface between the motor domain and CC1b, while in the T192Y-MD-NC-CC1 mutant, the bulky aromatic sidechain of Y192 protrudes from the motor domain (Fig 6D and 6E). The T192Y mutation is therefore likely to introduce a steric hindrance that could prevent CC1b from interacting with the motor domain (Fig 6G), which would ultimately activate the motor for processive movement.

To address whether the regulation of UNC-104 and KIF13B is conserved, we further created the *unc-104(L428A)* mutant using the CRISPR/Cas9 technique. The L428 is located in the CC1 region and corresponding to the L415 site on KIF13B (Fig 6A). Based on the crystal structure, L415 in KIF13B mediates the direct interaction with T192 in the motor domain, and therefore L428 in UNC-104 is predicted to make the contact with C184 in the motor domain (Fig 6E). Similar to the C184Y mutant, the L428A mutation in the MD-NC-CC1 fragment of UNC-104 also showed a significant enhancement in the ATPase activity compared to the wild-type protein (S6B Fig), suggesting the conserved role of L428 for UNC-104 autoinhibition. In *unc-104(L428A)* mutant animals, ectopic synaptic vesicles appear in the distal tip and dendrite region of DA9 (Fig 6H–6J). Kymograph analysis in the dorsal proximity region further showed that parameters associated with anterograde movements were significantly increased for UNC-104^L428A^ compared to wild-type UNC-104 (S8A–S8D Fig). Additionally, we created GFP knock-in line for UNC-104(L428A). Similar to C184Y::GFP, the L428A::GFP accumulates on neuronal processes (Fig 6L). In contrast to the cell body localization of wild type UNC-104::GFP, the L428A::GFP is generally absent from neuronal cell bodies (Fig 6K and 6L). Thus, mutations in the CC1 region, which disrupt the motor-CC1 interactions, may also lead to constitutively activating UNC-104 motor *in vivo*.

### New point mutations located at the MD-CC1 interface lead to gain of function of UNC-104

**Fig 7:**
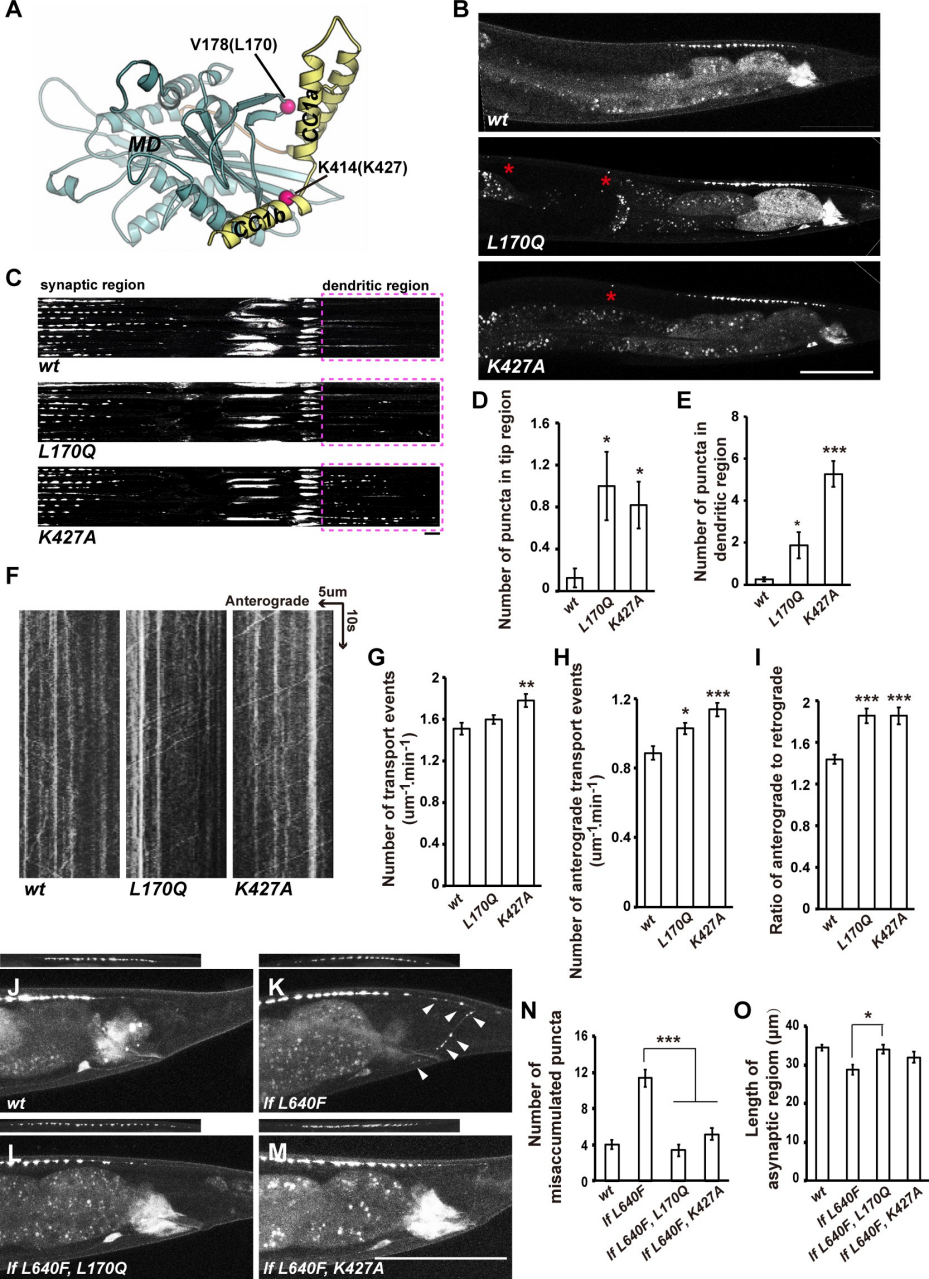
Mutations of the MD-CC1 interface lead to gain of function of UNC-104. (A) Mapping of the mutation sites on the structure of KIF13B MD-NC-CC1. The L170Q and K427A mutations are indicated. (B) Puncta formed by GFP::RAB-3 (driven by P*itr-1*) appear in the tip region of the DA9 neuron in *unc-104(L170Q)* and *unc-104(K427A)* animals (asterisks). Scale bar represents 50 μm. (C) GFP::RAB-3 puncta appear in the dendritic region of DA9 in *unc-104(L170Q)* and *unc-104(K427A)* animals. Line scan images of DA9 neurons. Ten DA9 neurons from independent animals were scanned and aligned. Scale bar represents 5 μm. Dashed boxes indicate the dendritic region. (D and E) The numbers of GFP::RAB-3 puncta in the tip region (D) and dendritic region (E).* P<0.05, *** P<0.001, one-way ANOVA with Tamhane’s T2 test. Mean ± SEM, n> = 20 for each genotype. (F) Representative kymograph images showing transport events of GFP::RAB-3 particles in the dorsal proximity region of DA9 neurons in wild type (*wt*), *unc-104(L170Q)* and *unc-104(K427A)* animals. Time and length are on the y axis and x axis, respectively. (G) The total number of transport events (anterograde plus retrograde) was measured by counting the number of GFP::RAB-3 particles that passed through a 1-μm zone during the 1-min monitoring period. (H) The number of anterograde movement events was measured by counting the number of GFP::RAB-3 particles moving in the anterograde direction through the 1-μm zone during the 1-min monitoring period. (I) The ratio of anterograde movements to retrograde movements. * P<0.05, ** P<0.01, *** P<0.001, one-way ANOVA with Tukey’s test. Mean ± SEM, N = 30 for each genotype. (J-M) The abnormal synaptic accumulation defect in *unc-104(lf L640F)* could be suppressed by L170Q or K427A mutation on UNC-104. The synaptic region is shown on the top. Scale bar represents 25 μm. (N) Quantification of the misaccumulated GFP::RAB-3 puncta in the asynaptic and commissure regions. (O) Quantification of the length of the asynaptic region. *P<0.05, ***P<0.001, one-way ANOVA with Tamhane’s T2 test. Mean ± SEM, N> = 20 worms for each genotype.

Based on the structural analysis and the sequence similarity between KIF13B and UNC-104/KIF1A, we further predicted that the L170 site in the motor domain and the K427 site in CC1 (corresponding to V178 and K414 in KIF13B) may also be involved in the direct autoinhibitory contact between the motor domain and CC1 of UNC-104/KIF1A (Figs 6A and 7A). Hence, we used CRISPR/Cas9 to create two new *unc-104* alleles, *xd407* and *xd408*. The *unc-104(xd408)* mutant gene encodes an UNC-104 motor containing the L170Q mutation and the *unc-104(xd407)* allele encodes an UNC-104 motor containing the K427A mutation. To test whether *xd408(L170Q)* and *xd407(K427A)* behave as gain-of-function alleles of *unc-104*, we performed the following experiments. Firstly, we examined the synaptic vesicle distribution in DA9 neurons. We found that both the L170Q and K427A mutations lead to ectopic GFP::RAB-3 puncta appearing in the tip and dendritic region of DA9 neurons (Fig 7B–7E). We further measured the axonal transport in the dorsal proximity region of DA9 neurons. In comparison with the wild type, movements of GFP::RAB-3 puncta, particularly anterograde movements, were also significantly increased in UNC-104^L170Q^ or UNC-104^K427A^-containing animals (Fig 7F–7I). Secondly, we created the *unc-104(lf 640F*, *L170Q)* and *unc-104(lf 640F*, *L170Q)* mutant animals using CRISPR/Cas9 genome editing. We found that the uneven distribution of DD synaptic vesicle clusters in *unc-104(lf 640F*) could be rescued by L170Q or K427A mutation (S8E–S8H Fig). Thirdly, we created double mutants between *arl-8* and *xd408(L170Q)* or *xd407(K427A)*. We found that the synaptic vesicle transport defect of *arl-8* in DA9 neurons could be efficiently suppressed by *xd408(L170Q)* or *xd407(K427A)* mutant (S8I–S8N Fig). Furthermore, the mis-accumulated synaptic puncta in *unc-104(lf 640F)* mutant DA9 could be suppressed by L170Q or K427A mutation (Fig 7J–7O). Taken together, the CC1/motor domain-mediated autoinhibition is crucial for UNC-104/KIF1A function *in vivo*.

## Discussion

Till now, two kinds of intramolecular interactions could mediate UNC104/KIF1A autoinhibition. One is the intramolecular interaction between CC2 and FHA domains, another is NC-CC1 interaction (Introduction). Here, we revealed the third intramolecular interaction, which is mediated by the motor domain and the CC1 domain. With extensive genome-editing based genetic analysis, we demonstrated the complex allelic relationship between different loss-of-function and gain-of-function alleles of *unc-104*. Combined with crystal structure and *in vitro* ATPase activity measurements, we further revealed that the CC1/motor intramolecular interaction could mediate the autoinhibition of UNC-104/KIF1A. Unlike the transgenic overexpression of UNC-104, we also showed that the endogenous wild type UNC-104 motors are diffusely distributed in cell bodies and neuronal processes. In contrast, gain-of-function motors are generally lacking from cell bodies. Instead, they accumulate on neuronal processes, particularly at the neuronal terminals.

### Molecular basis of the gain-of-function alleles of *unc-104*

Genetic screens have been widely used to explore the molecular, cellular and developmental mechanisms underlying various biological processes. In fact, UNC-104/KIF1A, the axonal transport kinesin for synaptic vesicles and the founding member of the kinesin-3 family, was originally identified through a locomotion behavior screen in *C*. *elegans* three decades ago [1]. Through visual screens, we firstly isolated two loss-of-function alleles of *unc-104*. The *xd53(L640F)* mutation resides in CC2, while the *xd130(G1092E)* mutation is located in the flexible linker region between CC3 and the C-terminal PH domain. Further modifier screens identified that disrupting the CC1/motor mediated autoinhibition could suppress the reduction of UNC-104 activity caused by different mutations (L640F and G1092E) implying that the uninhibited state of the MD-NC-CC1 region is sufficient to drive UNC-104/KIF1A motor activation. Consistent with this assumption, the structural analysis demonstrated that the T192Y-MD-NC-CC1 mutant adopts an active dimeric conformation (Fig 6). The loss-of-function allele *e1265* contains a D1497N mutation on the C-terminal PH domain. Intriguingly, the synaptic vesicle mis-accumulation defect of *e1265(D1497N)* could not be suppressed by gain-of-function mutation C184Y, G421E or L410F. This observation supports the notion that cargo binding is a prerequisite for the cargo transport activity of a UNC-104 motor. Noticeably, two intragenic suppressors of the mutation in the PH domain have been identified. It appears that while loss of cargo binding reduces motor levels, these two suppressors could restore the UNC-104 level and cargo binding [23]. It will be interesting to test whether the enhancement of cargo binding could influence motor activation as well.

The gain-of-function motor UNC-104^C184Y^ tends to accumulate on neuron processes. In PLM neurons, the UNC-104^C184Y^ motor is enriched at the terminal region of the anterior branch but not the posterior end of PLM. Our previous studies demonstrated that actin is concentrated at the terminal region of the anterior PLM process, in which the PLM forms gap junctions with interneuron BDU [38]. Gap junction connections indeed exist at the terminals of some *C*. *elegans* neurons [29]. However, the distribution of UNC-104^C184Y^::GFP dots does not resemble the distribution pattern of gap junction connections. For PLN neuron, it is completely unclear whether any special cytoskeleton arrangement exists in the two places on which the UNC-104^C184Y^::GFP is accumulated. Hence, why UNC-104^C184Y^ motor specifically accumulates on above positions and whether the abnormal accumulation leads to any functional consequence of *C*. *elegans* neurons remain to be determined. Considering the synaptic vesicle transport is enhanced in *unc-104(C184Y)* mutants and the cell-body-distribution of UNC-104^C184Y^ motors is greatly reduced, it is a reasonable proposal that the C184Y mutation could lead to more motors in an activated state.

### Conservation of CC1/motor domain-mediated autoinhibition in the kinesin-3 family

Most KIF1A mutations causing the neuropathies are located within the conserved motor domain. A recent study showed that mutations in the motor domain could lead to hyperactivation of motor activity [24], but the underlying mechanisms remain to be explored. In this study, we identified a number of gain-of-function alleles of *unc-104* that caused hyperactive axonal transport of synaptic vesicles (Figs 2 and 3). Interestingly, the point mutations in these gain-of-function alleles are in the motor domain or CC1 and are specifically located in the inter-domain interaction interfaces in the compact MD-NC-CC1 monomer (Fig 6) [32]. Mutations of these sites in MD-NC-CC1 disrupted the autoinhibited state and restored the motor activity (Fig 6), which suggests that the CC1/motor domain-mediated autoinhibition characterized in KIF13B could apply to UNC-104/KIF1A. The L428 is located in the CC1 region and corresponding to the L415 site on KIF13B, which should mediate the direct interaction with C184 in the motor domain of UNC-104. The *unc-104(L428A)* mutant animals display typical gain-of-function features of *unc-104* and subcellular localization of UNC-104(L428A)::GFP resembles C184Y::GFP. Furthermore, when two new mutations in the UNC-104 motor domain or CC1 predicted to disrupt the inter-domain autoinhibitory interface were introduced into UNC-104 motor, the point mutations resulted in gain of UNC-104 function and hyperactive axonal transport *in vivo* (Fig 7). Taken together, CC1/motor domain-mediated autoinhibition is highly conserved in kinesin-3 motors.

### Potential implications for disease-related hyperactivation of UNC-104/KIF1A

The first cargo identified for UNC-104/KIF1A is synaptic vesicle precursors [1,2], and UNC-104/KIF1A misdelivery of synaptic vesicle precursors plays a prominent role in controlling the size and density of synapses [22,39]. In addition to synaptic vesicle precursors, KIF1A actually carries a variety of vesicles, including dense core vesicles and BACE-containing vesicle, which contribute to pre- and post-synaptic assembly, autophagic processes, and neuron survival [40]. Thus, mutations disrupting KIF1A motor motility are linked to severe neuronal diseases [7–9]. Notably, most of the disease-related mutations are enriched in the motor domain [40], which suggests that disruption of the normal motor domain function leads to disease pathogenesis. It was previously believed that the disease-associated mutations in the motor domain largely cause loss of function of KIF1A, thus disrupting the motor activity that is required for axonal transport. However, recent work demonstrated that some of these mutations are actually gain-of-function changes which can result in motor hyperactivity [24]. In this study, we also identified gain-of-function mutations in the motor domain that significantly enhance the motor activity of UNC-104/KIF1A for axonal transport (Figs 2 and 3). Further biochemical and structural analyses revealed that these mutations are located in the key sites of the autoinhibitory interface and can disrupt the CC1/motor domain-mediated autoinhibition (Fig 6), which may be the reason for the motor hyperactivation. It is possible that the hyperactivation associated with the disease-related mutants is caused by the disruption of CC1/motor domain-mediated autoinhibition. Thus, our work provides a potential mechanistic explanation for the role of disease-related mutations in motor hyperactivation.

## Materials and methods

### Worm strains and genetics

*C*. *elegans* strains are maintained on NGM plates seeded with OP50 *E*. *coli*. The genetic manipulation was performed as described [41]. Mutants and GFP knock-in strains for *unc-104* are: *xd408(L170Q)*, *xd359(C184Y)*, *xd475(L410F)*, *xd366(G421E)*, *xd407(K427A)*, *xd496(L428A)*, *xd53(lfL640F)*, *xd130(lfG1092E)*, *e1265(lf D1497N)*, *xd53 xd408(lfL640F*, *L170Q)*, *xd53 xd359(lfL640F*, *C184Y)*, *xd53 xd475 (lfL640F*, *L410F)*, *xd53 xd366 (lfL640F*, *G421E)*, *xd53 xd407(lfL640F*, *K427A)*,*xd130 xd359 (lfG1092E*, *C184Y)*, *xd130 xd475(lfG1092E*, *L410F)*, *xd130 xd366 (lfG1092E*, *G421E)*, *xd500 xd359(lf D1497N*, *C184Y)*, *xd501 xd475(lf D1497N*, *L410F)*, *xd502 xd366(lf D1497N*, *G421E)*, *xdKi3(unc-104*::*gfp knock-in)*, *xdKi6(C184Y*::*gfp knock-in)*, *xdKi60(L428A*::*gfp knock-in)*,*xdKi4(lf L640F*::*gfp knock-in)*, *and xdKi61(lf L640F*, *C184Y*::*gfp knock-in)*. Other strains are: *xdIs164*(P*unc-25*::GFP::RAB-3*)*,*xdEx968 (Pmec-7*::*mCherry)* and *xdEx2678 (Plad-2*::*mCherry)*. *wyIs85(Pitr-1*::*GFP*::*RAB-3)* was provided by Kang Shen (Stanford University). The *unc-104 (xd53)* and *unc-104 (xd130)* worms were isolated from *xdIs164* worms treated with EMS. The*xd53 xd359(lfL640F*, *C184Y)*, *xd53 xd475 (lfL640F*, *L410F)*, *xd53 xd366 (lfL640F*, *G421E)* worms were obtained from an EMS-based genetic screen using*unc-104(xd53);xdIs64* strain. Briefly, the distribution of synaptic vesicles in F2 progeny was examined under a fluorescence microscope, and suppressor mutant animals were recovered to produce progeny. A total of 7,200 mutagenized haploid genomes were screened and total eight suppressors were isolated from this screen.

### DNA constructs and CRISPR/Cas9

*unc-104*cDNAs were amplified by PCR and cloned into dpSM vectors (from Dr. Kang Shen, Stanford University). psc325 vector (from Dr. Yishi Jin, UCSD)contains the *unc-25* promoter. Transgenic animals were made by standard microinjection procedures. In general, plasmid DNAs of interest were used at 1–50 ng/μl, and the co-injection marker was *Pmyo-2*::*RFP* or *Podr-1*::*RFP* or *Pord-1*::*GFP*. All transgenic strains were crossed to N2 wild type 4 times before use. The CRISPR/Cas9-mediated genome editing strains were generated by SunyBiotech and were verified with DNA sequencing. All strains were outcrossed with N2 for 4 times before use. The detailed information is included in S2 Table.

### Imaging collection and quantification

Animals were mounted on 2.5% agar pads in M9 buffer containing 1% 1-phenoxy-2-propanoland. Fluorescence photographs were taken using a Leica SP8 confocal microscope unless specifically indicated. The GFP::RAB-3 fluorescence images of DD/VD neurons were taken at the young adult stage. The GFP::RAB-3 fluorescence images of the DA9 neuron were taken at the first day of adulthood. Straightened dorsal cord images were obtained using ImageJ software. The lengths of the synaptic region and the asynaptic region were measured by ImageJ. For each genotype, more than 20 worms were imaged.

### Kymograph analysis

Time-lapse images of GFP::RAB-3 were taken at the L4 stage, using an inverted Zeiss Axion Observer Z1 microscope equipped with a Plan-Apochromat 63x/1.4 objective, an Andor EM-CCD digital camera, and a Yokogawa CSU-X1 spinning disk system. 201 frames were taken for each movie with a speed of 6 frames per second and an exposure time of 160 ms. For movie acquisition, worms were mounted on 2.5% agar pads and anesthetized with 1-phenoxy-2-propanol. Movies were analyzed using ImageJ. The “register” plugin was used for movie correction. Subsequently, “segmented line” was used to select the region for kymograph analysis. Finally, kymographs were constructed with the “kymograph” plugin. For each genotype, 30 worms were imaged.Each contiguous line with a constant slope was scored as a single transportevent and its run length (distance moved along the longaxis of the neurite) were calculated. Run length is defined as a singlemotility occurrence typically right after a pause or a reversal and,vice versa, ends when the motor is again pausing or reversing.

### Fluorescence recovery after photobleaching

The FRAP experiments were performed on the young adult animals using a laser scanning confocal microscope (LSM980, Zeiss) equipped with a Plan-Apochromat 63x/1.4 NA oil immersion. For bleaching, worms were mounted on 2.5% agar pads and anesthetized with 1-phenoxy-2-propanol. Photobleaching, image acquisition, and image analysis were performed on ZEN system. Image acquisition was conducted for regions with 792 x 792 pixels (0.09μm/pixel) using 488-nm semiconductor lasers at 10% power with the pinhole diameter 56μm. Prior to the bleaching, 3 frames of images were acquired. Photobleaching was performed on a rectangle region with size 55 x 22 pixels using 488-nm lasers at 20% power for 250ms. After photobleaching, images were collected till no further recovery was detected. The fluorescence recovery of the bleached region was calculated as follows. Firstly, average fluorescence intensities within the bleached region and non-bleached regions (as the control value) were measured. Second, the lowest intensity value (immediately after bleaching) within the photo bleaching region was subtracted from all measurements. The average intensities of the 3 measurements that precede the bleaching were then determined, establishing a pre-bleach value; all intensities were normalized by dividing by that value. The halftime of recovery is the time point where the fluorescence recovered to half of the maximum recovery.

### Statistical analysis

To compare multiple groups, one-way ANOVA was used with an appropriate multiple comparisons *post hoc* test (the test used is stated in each figure legend). * P<0.05; ** P<0.01; *** P<0.001.

### Worm motility assay

Worms were assessed for Unc phenotypes and locomotion defects by examining their motility on NGM plates. Young adult worms were transferred to new plates and movement was recorded under a dissection microscope (Nikon) equipped with a HQ CCD camera (Aoka).For velocity assay, movements were recorded at 1–2 frames/s. The obtained movements were tracked using a custom programmed ImageJ’s “multitracker” plugin.

### High-pressure freezing electron microscopy

Young adult worms were cryofixed on a Leica Microsystems HPM 100 and frozen in liquid nitrogen. After high-pressure freezing, the samples were washed 4 times in acetone and stained with 1% uranyl acetate for 1 hr. The infiltration was performed with increasing concentrations of Embed 812. Finally, samples were embedded in fresh resin at 60°C for 3 days. Thin sections (60 nm) were produced with a diamond knife (Diatome) on an ultramicrotome (Ultracut UCT, Leica Microsystems). Serial sections were pictured with a JEM-1400 electron microscope at 80 kV. Pictures were recorded using a Gatan832 4k × 2.7k CCD camera.

### Protein extraction and western blot analysis

Worms were collected and washed in M9 buffer. Lysis buffer (50 mM Tris·HCl, 150 mM NaCl, 1% Nonidet P-40, Roche cocktail protease) was added and worms were disrupted with a Dounce homogenizer (Cheng-He Company, Zhuhai, China) on ice for 15 min. Debris was removed by centrifuging at 12000 rpm for 15 minutes at 4°C. For western blotting, protein samples were boiled and separated in an 8% SDS-PAGE gel. After transfer, the nitrocellulose membranes (Pall Life Sciences) were incubated with 5% dry milk and probed with an anti-GFP antibody (1:5000 dilution, Abcam). The signals were visualized with a Supersignal West Pico Substrate kit (Pierce). The stained bands were analyzed by ImageJ Software. Graphical data are presented as mean ± SEM. n = 3 independent experiments.

### Protein expression and purification

DNA sequences encoding rat KIF13B MD-NC-CC1 and various mutants were cloned individually into the pET32a vector with a carboxyl His_6_-tag. Point mutations were introduced using the standard PCR-based mutagenesis method and confirmed by DNA sequencing. Recombinant proteins were expressed in *Escherichia coli* BL21 (DE3) host cells at 16°C. The His_6_-tagged fusion proteins were purified by Ni^2+^-Sepharose 6 Fast Flow (GE healthcare) affinity chromatography followed by size-exclusion chromatography (Superdex-200 26/60, GE Healthcare) in buffer containing 50 mM Tris-HCl, pH 7.5, 150 mM NaCl, 2 mM MgCl_2_, 1 mM EGTA, 1 mM DTT. For analytical gel-filtration analysis, protein samples were concentrated to ~2.5 mg/ml and loaded onto Superdex-200 10/300 GL columns (GE Healthcare).

### Microtubule-stimulated ATPase assay

Measurements of the microtubule-stimulated ATPase activities of KIF13B MD-NC-CC1 and various mutants were performed using the HTS Kinesin ATPase Endpoint Assay Biochem Kit (Cytoskeleton, Inc.). Briefly, all the measurements were based on the malachite green phosphate assay to probe inorganic phosphate generated during the reaction. A standard curve of phosphate was made to estimate the amount of phosphate generated. The basal ATPase activities (without microtubules) of these KIF13B fragments were also measured using the same method. Each protein sample had three replicates and each measurement was repeated at least three times independently. The kinesin-1 heavy chain (KHC) supplied in the Kit was used as the control.

### Crystallization, data collection and structural determination and refinement

Crystals of the KIF13B T192Y-MD-NC-CC1 mutant (~25 mg/ml) were obtained in 0.1 M Bis-Tris propane, pH 8.2, 30% (w/v) PEG 20000 using the sitting drop vapor diffusion method at 16°C. Before being flash-frozen in liquid nitrogen, crystals were soaked in the mother liquor supplemented with 15% (v/v) ethylene glycol for cryo-protection. The diffraction datasets were collected at the beamline BL17U1 of the Shanghai Synchrotron Radiation Facility with a wavelength of 0.989 Å at 100K [42]. Datasets were integrated and scaled with HKL2000 [43]. The structure was determined by the molecular replacement method using the motor domain of KIF13B (PDB code: 5ZBR) as the searching model with Phaser [44]. Additional missing residues were manually modeled into the structure according to the *2Fo-Fc* and *Fo-Fc* electron density maps using COOT [45]. The structure was further refined and validated with PHENIX [46]. The statistics for the data collection and structural refinement are summarized in S1 Table.

## Supporting information

S1 Fig The axonal transport defect in *unc-104(xd53)*. (A and B) The distribution of GFP::RAB-3 puncta (green) in wild type (*wt*) and *unc-104(xd53)*. White arrows indicate DD neurons. White brackets indicate regions lacking GFP::RAB-3 puncta. The schematic drawings on the right show the synaptic vesicle distribution in DD neurons. (C and D) EM images of dorsal synapses in *wt* and *unc-104(xd53)*. M, muscle. White arrows indicate active zones. Scale bar represents200 nm. (E-H) The distribution of P*unc-25*::GFP::RAB-3 (green) puncta in *wt*, *unc-104(lfL640F*, *C184Y/lfL640F)*, *unc-104(lfL640F*, *L410F/lfL640F)*, and *unc-104(lfL640F*, *G421E/lfL640F)* worms. Scale bar represents 50 μm.
(TIF)

S2 Fig The locomotion defect of *unc-104(lf G1092E)* is suppressed by the C184Y, L410F or G421E mutation of UNC-104. (A-D) Snapshots were taken at 10 s, 5 min and 20 min of *unc-104(lfG1092E)*, *unc-104(lfG1092E*, *C184Y)*, *unc-104(lfG1092E*, *L410F)*, and *unc-104(lfG1092E*, *G421E)* worms. Dashed circles indicate the spots on which 15 worms of each genotype were placed. Scale barrepresents1 mm. (E) Quantification of the velocity in various genotypes. ***P<0.001, one-way ANOVA with Tamhane’s T2 test. Mean ± SEM, N> = 15 worms for each genotype.
(TIF)

S3 Fig The synaptic vesicle transport defect of *unc-104(lf D1497N)* could not be suppressed by the C184Y, L410F or G421E mutation on UNC-104. (A) Schematic drawing of the domain organization of UNC-104 motor protein. The mutation sites of *xd359(C184Y)*, *xd475(L410F)*, *xd366(G421E)* and *e1265(lf D1497N)*. *xd359*, *xd475* and *xd366* are indicated. (B) The even distribution of GFP::RAB-3 puncta on the dorsal cord is not restored in *unc-104(lf D1497N*, *C184Y)*, *unc-104(lf D1497N*, *L410F)* and *unc-104(lf D1497N*, *G421E)* animals.
(TIF)

S4 Fig The synaptic vesicle transport defect of *unc-104(lf L640F)* could be suppressed by the C184Y or G421E mutation on UNC-104. (A-C) The distribution of GFP::RAB-3 puncta (green) driven by P*unc-25* in *xd359 (C184Y)* (A), *xd475 (L410F)* (B) and *xd366 (G421E)* (C). Scale bar represents 50 μm. (D) The schematic drawing shows the synaptic vesicle distribution of DD neurons. (E-F) The abnormal synaptic accumulation defect in *unc-104(lf L640F)* could be suppressed by C184Y (G) or G421E (H) mutation on UNC-104. (I)Quantification of the misaccumulated GFP::RAB-3 puncta in the asynaptic region and commissure region. (J) Quantification of the length of the asynaptic region. **P<0.01; NS, not significant. One-way ANOVA with Tamhane’s T2 test. Mean ± SEM, N> = 20 worms for each genotype.
(TIF)

S5 Fig The expression level of wild-type and UNC-104^C184Y^ mutant motors. (A) Western blot of worm lysates probed with a GFP antibody to detect the expression level of wild-type and UNC-104^C184Y^ motors. (B) Quantification of the expression level of the motor proteins in (A). Mean ± SEM, two-tailed paired Student’s t test. NS, not significant. (C) Quantification of the percentage of the worms with GFP accumulation on the anterior tip region of PLM neuron. Mean ± SEM, two-tailed unpaired Student’s t test. Total 300 worms were examined for each genotype. N = 6. (D) Quantification of the percentage of the worms with normal PLM morphology. Mean ± SEM, two-tailed unpaired Student’s t test. About 130 worms were examined. N = 3. (E) Quantification of the percentage of the worms with normal synapse formation. Mean ± SEM, two-tailed unpaired Student’s t test. 250 worms were examined. N = 5. ***P<0.001; NS, not significant.
(TIF)

S6 Fig Structure of the T192Y-MD-NC-CC1mutant. (A) Analytical gel-filtration analysis of wild type and various mutants of the MD-NC-CC1 region of KIF13B (T192Y, L397F and T408E). (B) Microtubule-stimulated ATPase activity of the UNC-104 MD-NC-CC1 fragment containing the C184Y, L410F, G421E, or L428A mutation. Bars represent Mean ± SD. n = 4 independent experiments, * P<0.05, one-way ANOVA with LSD test. (C) Structure of the T192Y-MD-CC1 mutant fragment of KIF13B. The inset shows the omit electron-density maps of ADP and Mg^2+^ (contoured at 1.5σ level).
(TIF)

S7 Fig The synaptic vesicle transport defect of *arl-8* mutant could be suppressed by the C184Y or G421E mutation on UNC-104. (A-E) The GFP::RAB-3 puncta distribution in wild type, *arl-8*, *arl-8;unc-104(C184Y)*,*arl-8;unc-104(L410F)*and *arl-8;unc-104(G421E)* worms. The synaptic region is shown on the top. Scale bar represents 25 μm. (F) Quantification of the misaccumulated GFP::RAB-3 puncta in the asynaptic region and commissure region. (G) Quantification of the length of the asynaptic region. ***P<0.001, one-way ANOVA with Tukey test. Mean ± SEM, N> = 20 worms for each genotype.
(TIF)

S8 Fig The L428A, L170Q and K427A mutations of the MD-CC1 interface lead to gain of function of UNC-104. (A) Representative kymograph images showing transport events in wild type (*wt*) *unc-104(L428A)*. Time and length are on the y axis and x axis, respectively. (B) The number of stable GFP::RAB-3 particles in a 1-μm section within 1 min. (C) The number of anterograde transport events. (D) The ratio of anterograde transport events to retrograde transport events. **P<0.01, ***P<0.001. Mean ± SEM, two-tailed paired Student’s t test. N = 30 worms for each genotype. (E-H) The even distribution of GFP::RAB-3 (driven by P*unc-25* promotor) puncta on the dorsal cord is restored in *unc-104(lf L640F*, *L170Q)* and *unc-104(lf L640F*, *K427A)* animals. (I-L) The synaptic vesicle transport defect of *arl-8* mutants could be suppressed L170Q or K427A mutation (M) Quantification of the misaccumulated GFP::RAB-3 puncta in the asynaptic region and commissure region. The synaptic region is shown on the top. Scale bar represents 25 μm. (N) Quantification of the length of the asynaptic region. ***P<0.001, one-way ANOVA with Tamhane’s T2 test. Mean ± SEM, N> = 20 worms for each genotype.
(TIF)

S1 Table Data collection and structural refinement statistics. (DOCX)

S2 Table Strains with genome-editing. (DOCX)
