# Supplementary figures and images for: Motor domain-mediated autoinhibition dictates axonal transport by the kinesin UNC-104/KIF1A
Source: PLoS Genet. 2021 Nov 29;17(11):e1009940. doi: 10.1371/journal.pgen.1009940 (PMC8659337; doi:10.1371/journal.pgen.1009940)

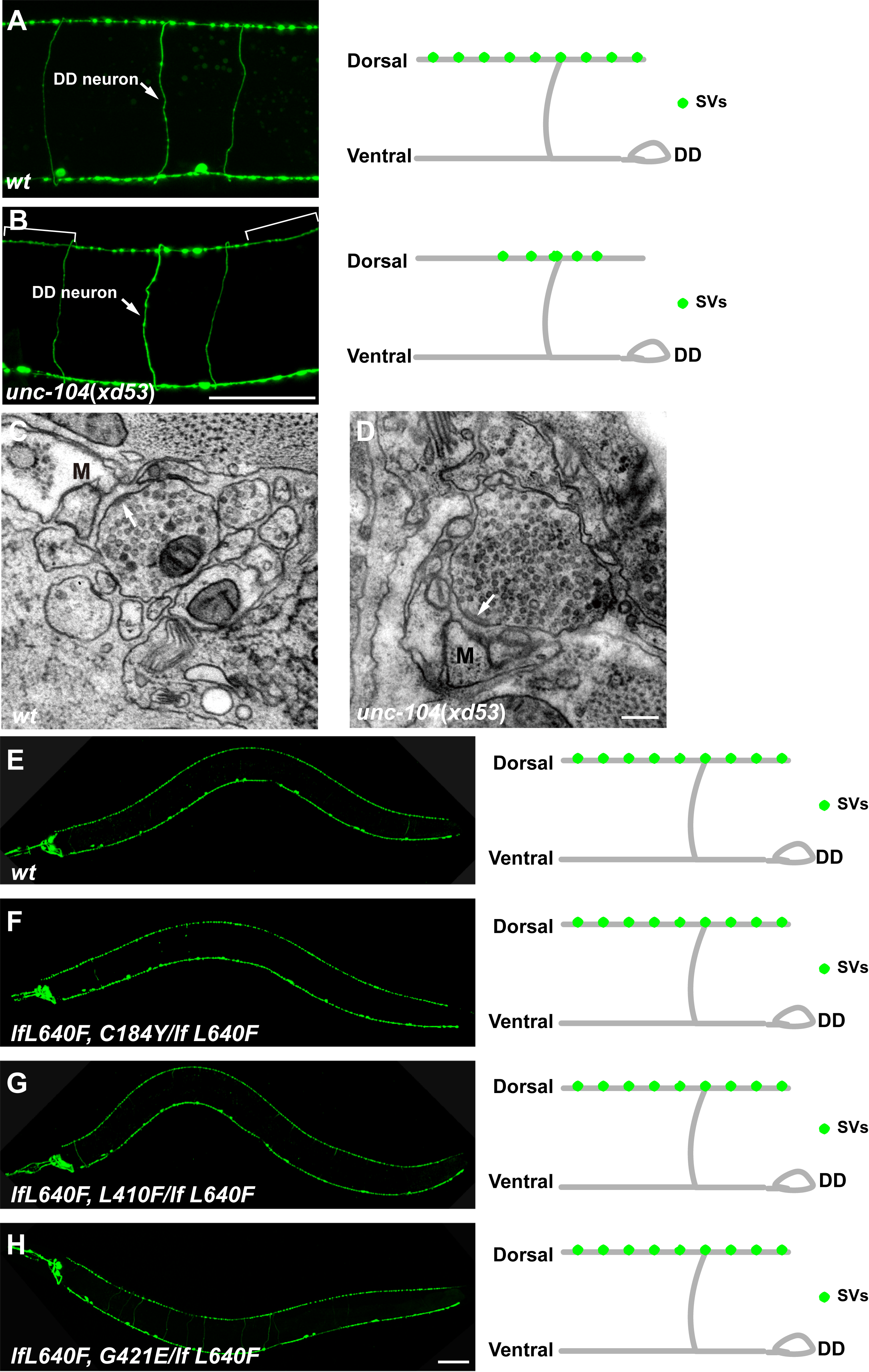

Supplement: S1 Fig — (A and B) The distribution of GFP::RAB-3 puncta (green) in wild type (wt) and unc-104(xd53). White arrows indicate DD neurons. White brackets indicate regions lacking GFP::RAB-3 puncta. The schematic drawings on the right show the synaptic vesicle distribution in DD neurons. (C and D) EM images of dorsal synapses in wt and unc-104(xd53). M, muscle. White arrows indicate active zones. Scale bar represents200 nm. (E-H) The distribution of Punc-25::GFP::RAB-3 (green) puncta in wt, unc-104(lfL640F, C184Y/lfL640F), unc-104(lfL640F, L410F/lfL640F), and unc-104(lfL640F, G421E/lfL640F) worms. Scale bar represents 50 μm. (TIF) [file pgen.1009940.s001.tif]

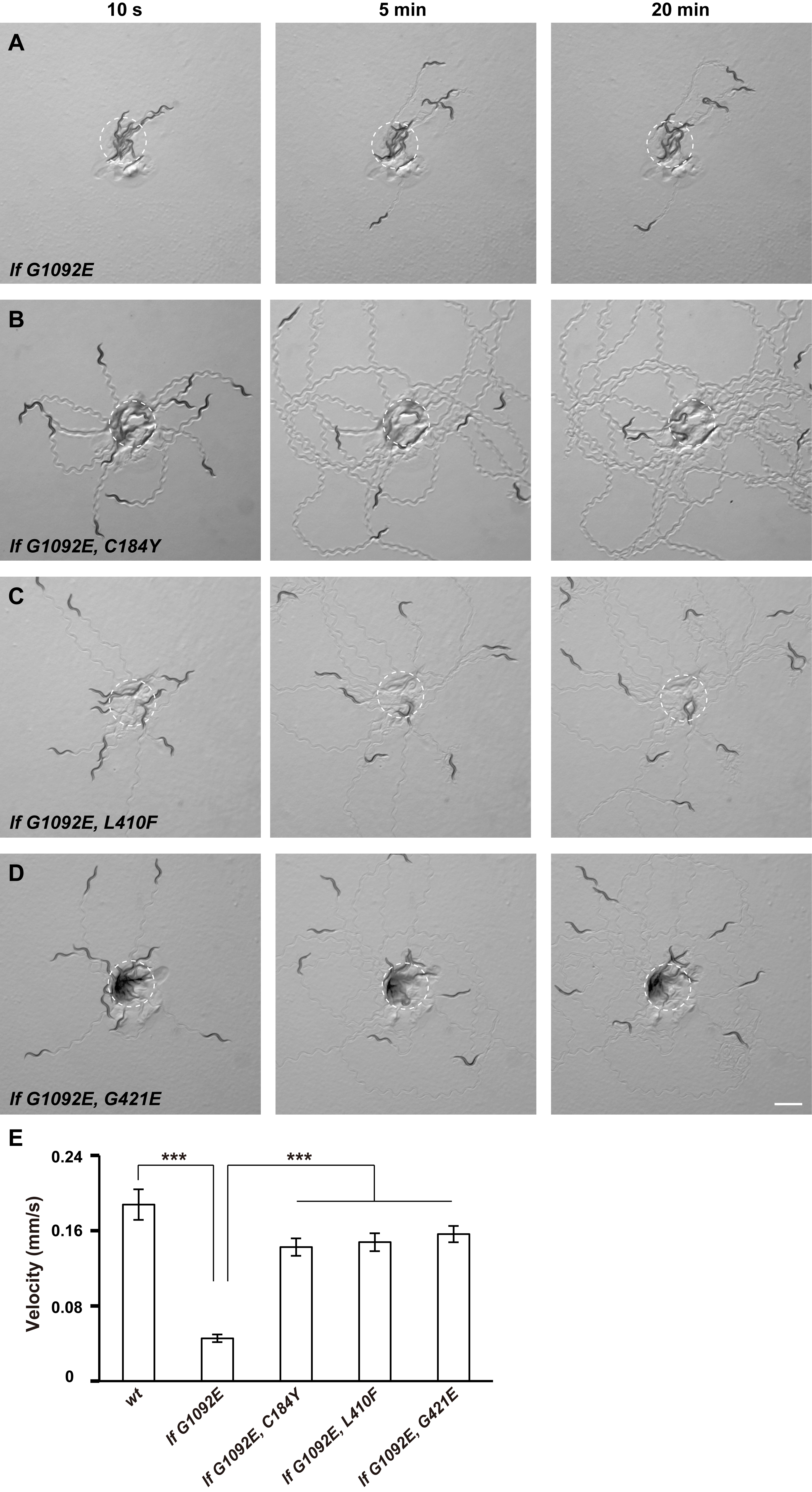

Supplement: S2 Fig — (A-D) Snapshots were taken at 10 s, 5 min and 20 min of unc-104(lfG1092E), unc-104(lfG1092E, C184Y), unc-104(lfG1092E, L410F), and unc-104(lfG1092E, G421E) worms. Dashed circles indicate the spots on which 15 worms of each genotype were placed. Scale barrepresents1 mm. (E) Quantification of the velocity in various genotypes. ***P<0.001, one-way ANOVA with Tamhane’s T2 test. Mean ± SEM, N> = 15 worms for each genotype. (TIF) [file pgen.1009940.s002.tif]

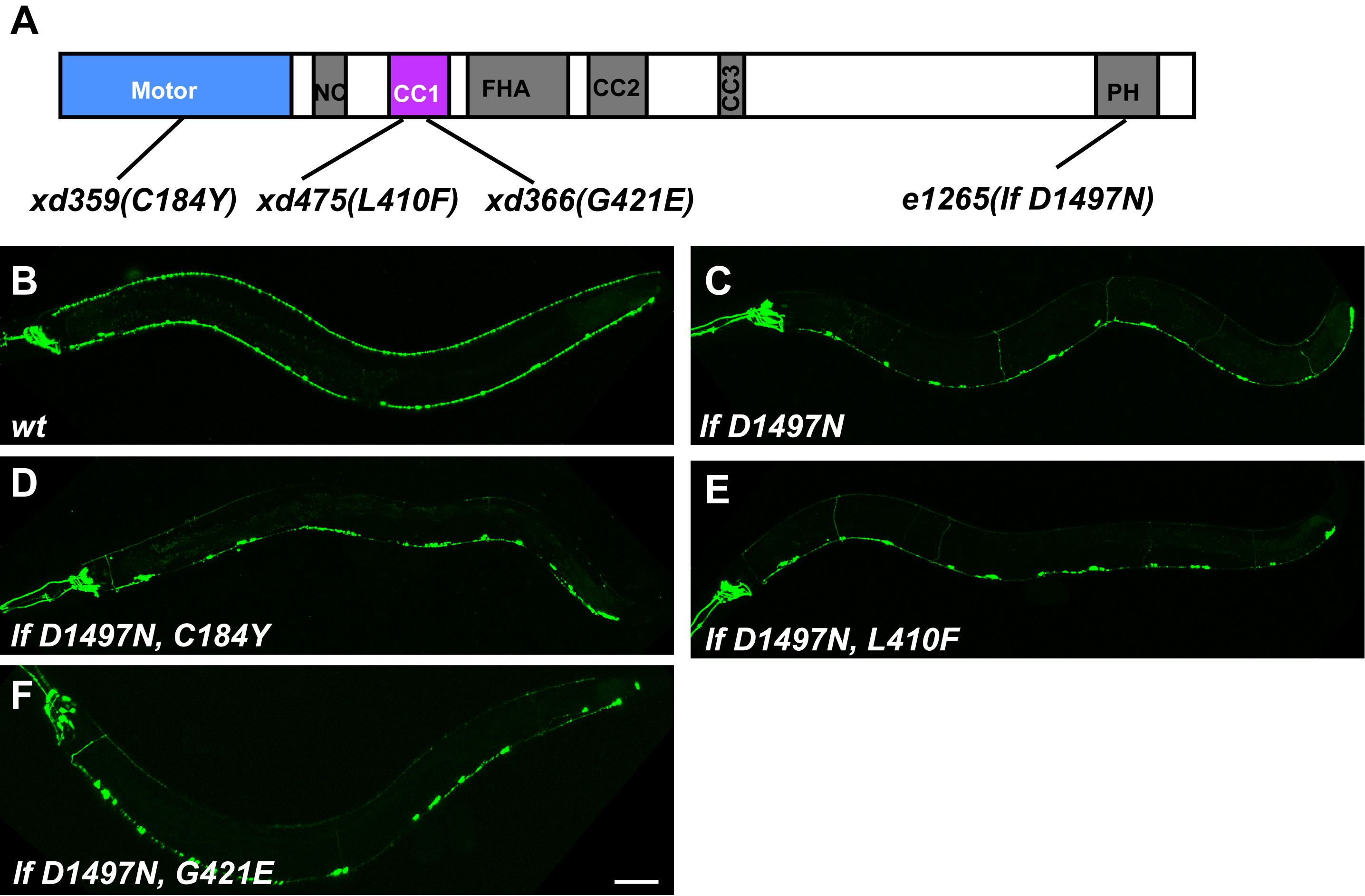

Supplement: S3 Fig — (A) Schematic drawing of the domain organization of UNC-104 motor protein. The mutation sites of xd359(C184Y), xd475(L410F), xd366(G421E) and e1265(lf D1497N). xd359, xd475 and xd366 are indicated. (B) The even distribution of GFP::RAB-3 puncta on the dorsal cord is not restored in unc-104(lf D1497N, C184Y), unc-104(lf D1497N, L410F) and unc-104(lf D1497N, G421E) animals. (TIF) [file pgen.1009940.s003.tif]

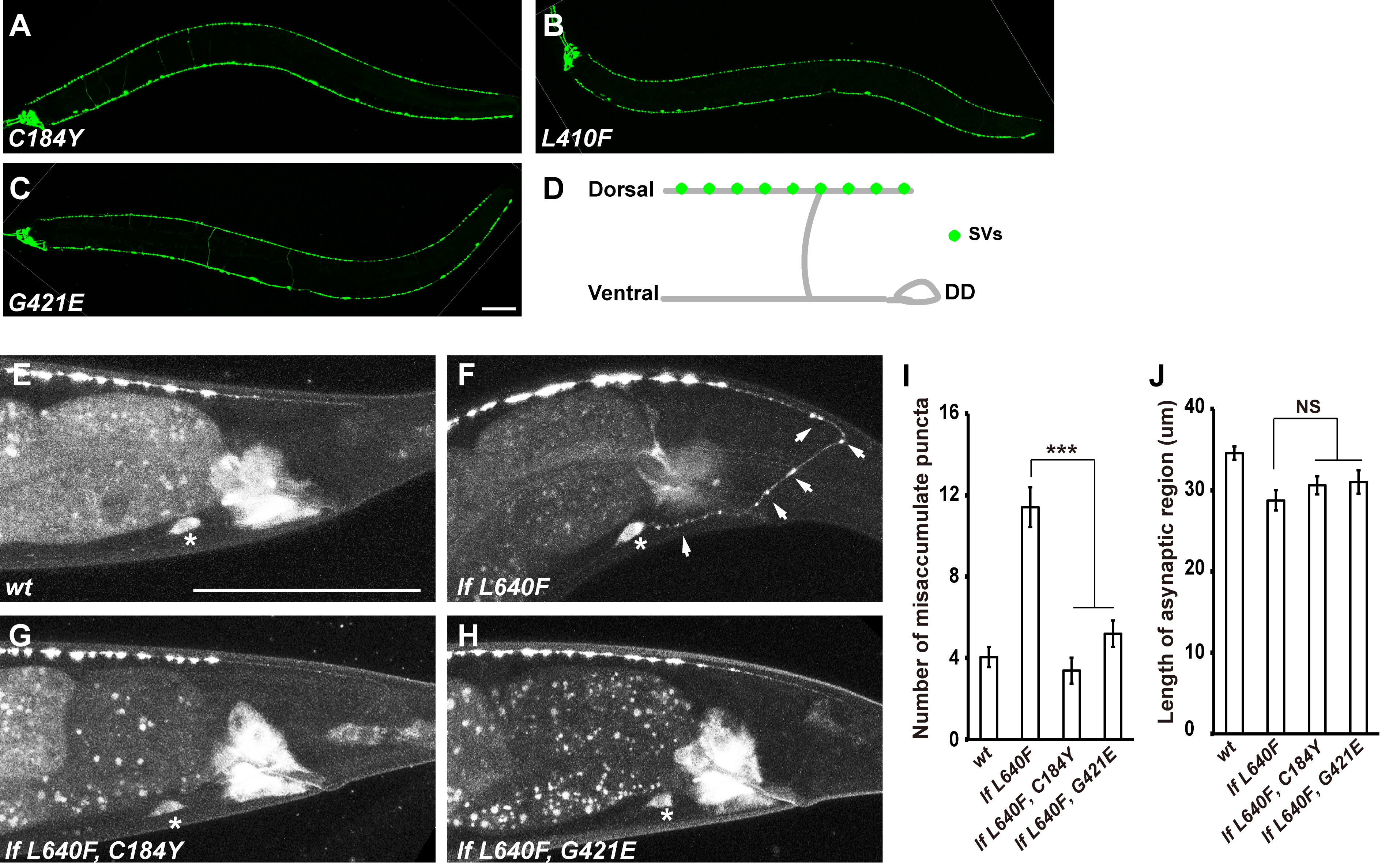

Supplement: S4 Fig — (A-C) The distribution of GFP::RAB-3 puncta (green) driven by Punc-25 in xd359 (C184Y) (A), xd475 (L410F) (B) and xd366 (G421E) (C). Scale bar represents 50 μm. (D) The schematic drawing shows the synaptic vesicle distribution of DD neurons. (E-F) The abnormal synaptic accumulation defect in unc-104(lf L640F) could be suppressed by C184Y (G) or G421E (H) mutation on UNC-104. (I)Quantification of the misaccumulated GFP::RAB-3 puncta in the asynaptic region and commissure region. (J) Quantification of the length of the asynaptic region. **P<0.01; NS, not significant. One-way ANOVA with Tamhane’s T2 test. Mean ± SEM, N> = 20 worms for each genotype. (TIF) [file pgen.1009940.s004.tif]

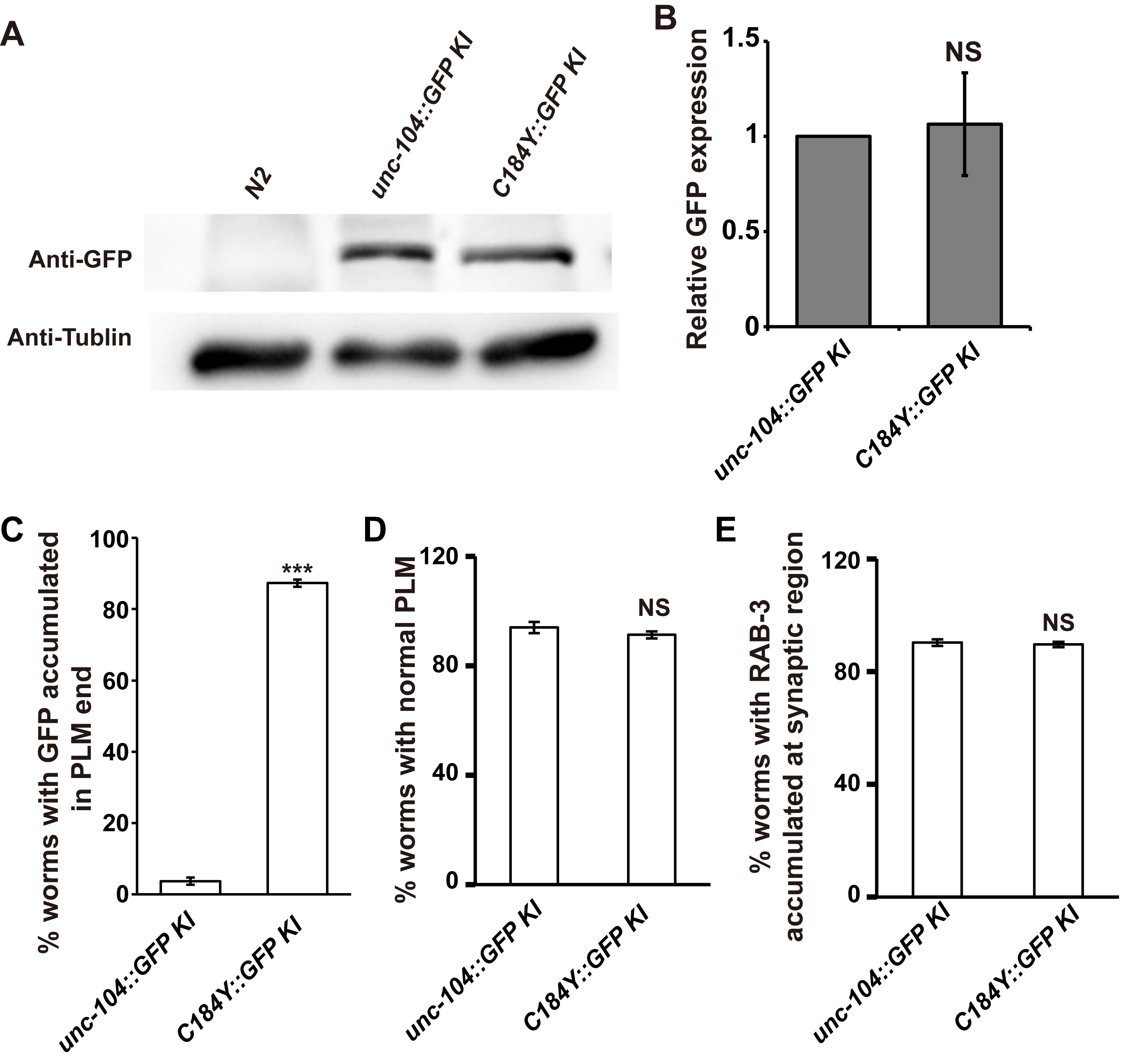

Supplement: S5 Fig — (A) Western blot of worm lysates probed with a GFP antibody to detect the expression level of wild-type and UNC-104C184Y motors. (B) Quantification of the expression level of the motor proteins in (A). Mean ± SEM, two-tailed paired Student’s t test. NS, not significant. (C) Quantification of the percentage of the worms with GFP accumulation on the anterior tip region of PLM neuron. Mean ± SEM, two-tailed unpaired Student’s t test. Total 300 worms were examined for each genotype. N = 6. (D) Quantification of the percentage of the worms with normal PLM morphology. Mean ± SEM, two-tailed unpaired Student’s t test. About 130 worms were examined. N = 3. (E) Quantification of the percentage of the worms with normal synapse formation. Mean ± SEM, two-tailed unpaired Student’s t test. 250 worms were examined. N = 5. ***P<0.001; NS, not significant. (TIF) [file pgen.1009940.s005.tif]

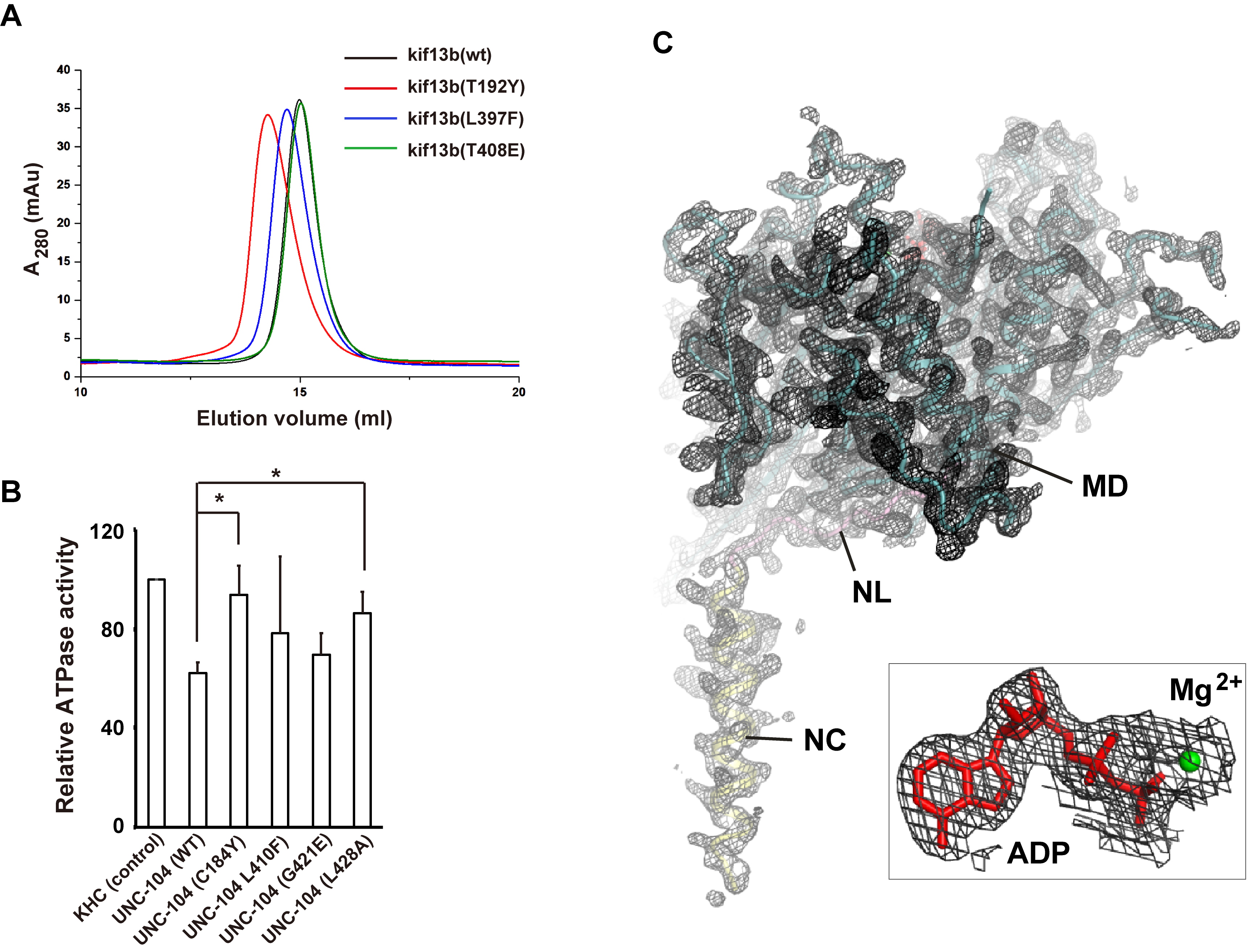

Supplement: S6 Fig — (A) Analytical gel-filtration analysis of wild type and various mutants of the MD-NC-CC1 region of KIF13B (T192Y, L397F and T408E). (B) Microtubule-stimulated ATPase activity of the UNC-104 MD-NC-CC1 fragment containing the C184Y, L410F, G421E, or L428A mutation. Bars represent Mean ± SD. n = 4 independent experiments, * P<0.05, one-way ANOVA with LSD test. (C) Structure of the T192Y-MD-CC1 mutant fragment of KIF13B. The inset shows the omit electron-density maps of ADP and Mg2+ (contoured at 1.5σ level). (TIF) [file pgen.1009940.s006.tif]

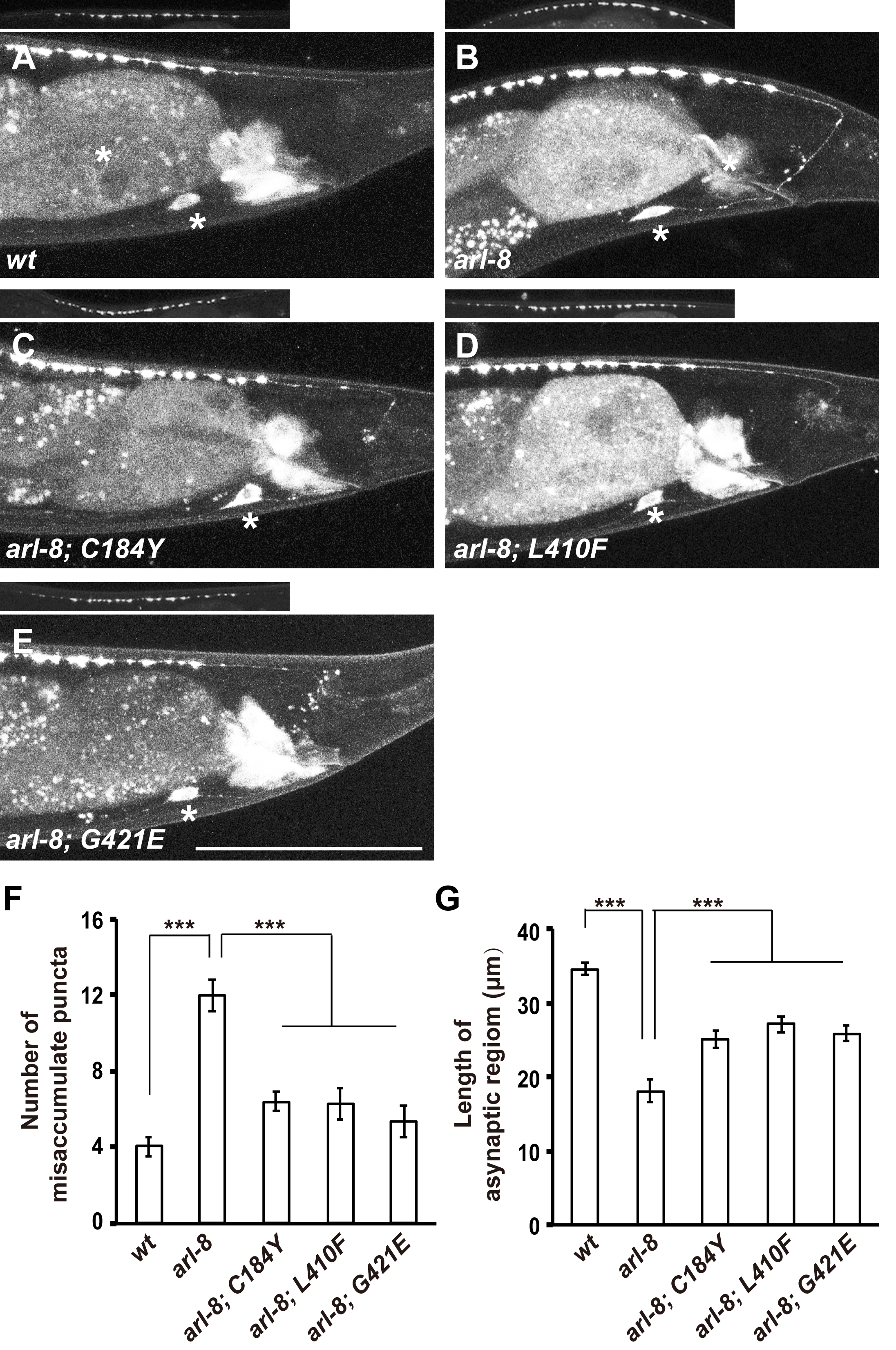

Supplement: S7 Fig — (A-E) The GFP::RAB-3 puncta distribution in wild type, arl-8, arl-8;unc-104(C184Y),arl-8;unc-104(L410F)and arl-8;unc-104(G421E) worms. The synaptic region is shown on the top. Scale bar represents 25 μm. (F) Quantification of the misaccumulated GFP::RAB-3 puncta in the asynaptic region and commissure region. (G) Quantification of the length of the asynaptic region. ***P<0.001, one-way ANOVA with Tukey test. Mean ± SEM, N> = 20 worms for each genotype. (TIF) [file pgen.1009940.s007.tif]

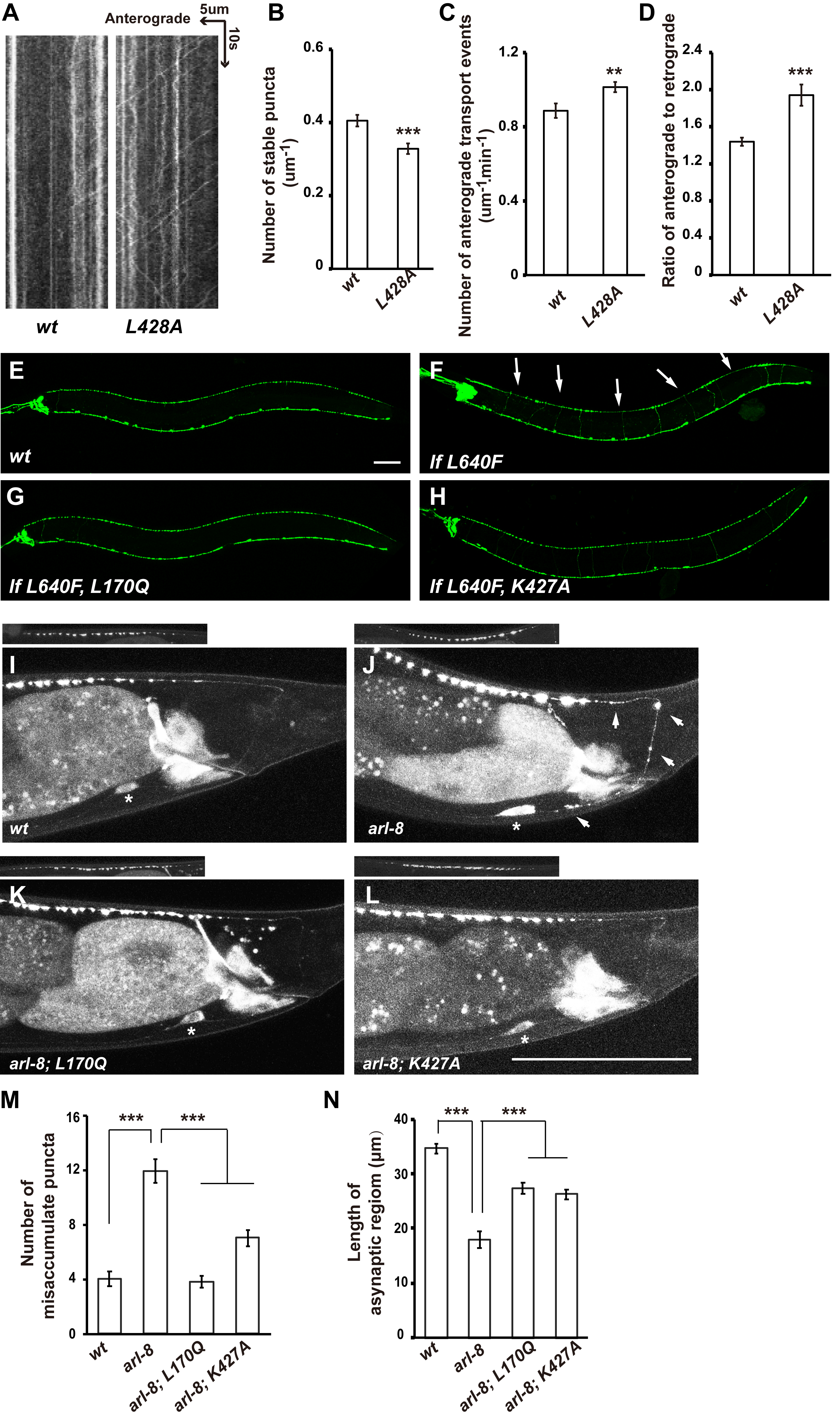

Supplement: S8 Fig — (A) Representative kymograph images showing transport events in wild type (wt) unc-104(L428A). Time and length are on the y axis and x axis, respectively. (B) The number of stable GFP::RAB-3 particles in a 1-μm section within 1 min. (C) The number of anterograde transport events. (D) The ratio of anterograde transport events to retrograde transport events. **P<0.01, ***P<0.001. Mean ± SEM, two-tailed paired Student’s t test. N = 30 worms for each genotype. (E-H) The even distribution of GFP::RAB-3 (driven by Punc-25 promotor) puncta on the dorsal cord is restored in unc-104(lf L640F, L170Q) and unc-104(lf L640F, K427A) animals. (I-L) The synaptic vesicle transport defect of arl-8 mutants could be suppressed L170Q or K427A mutation (M) Quantification of the misaccumulated GFP::RAB-3 puncta in the asynaptic region and commissure region. The synaptic region is shown on the top. Scale bar represents 25 μm. (N) Quantification of the length of the asynaptic region. ***P<0.001, one-way ANOVA with Tamhane’s T2 test. Mean ± SEM, N> = 20 worms for each genotype. (TIF) [file pgen.1009940.s008.tif]
