# Supplementary material for: Motor domain-mediated autoinhibition dictates axonal transport by the kinesin UNC-104/KIF1A
Source: PLoS Genet. 2021 Nov 29;17(11):e1009940. doi: 10.1371/journal.pgen.1009940 (PMC8659337; doi:10.1371/journal.pgen.1009940)
Supplement: S1 Table — (DOCX) [file pgen.1009940.s009.docx]

S1 Table: Data collection and structural refinement statistics

| *A. Diffraction data* | KIF13B MD-NC-CC1-T192Y |
| --- | --- |
| Space group | *P3221* |
| Wavelength (Å) | 0.9792 |
| Cell dimensions |  |
| a, b, c (Å) | 87.5, 87.5, 97.5 |
| α, β, γ (º) | 90, 90, 120 |
| Resolution (Å) | 32.6-2.30(2.34-2.30)a |
| Unique reflections | 19638(961) |
| I/σ(I) | 10.4(2.1) |
| Multiplicity | 10.8(9.7) |
| Completeness (%) | 100(100) |
| *B. Refinement* |  |
| Rworkb (%) | 18.2(22.9) |
| Rfreec (%) | 24.1(27.2) |
| Mean B factors (Å2) | 51.1 |
| R.m.s. deviationd |  |
| Bond length (Å) | 0.008 |
| Bond angles (º) | 0.998 |
| Ramachandran plot(%) |  |
| Favored region | 96.4 |
| Allowed region | 3.6 |
| Disallowed region | 0.0 |
| aThe values in parentheses refer to the highest resolution shell. | |
| b*Rwork* is the *Rfactor*for the working dataset. *Rfactor*=∑\|\|*Fo*\|-\|*Fc*\|\|/∑\|*Fo*\| where \|*Fo*\| and \|*Fc*\| are observed and calculated structure factor amplitudes respectively. | |
| c*Rfree* is the cross-validation *Rfactor* computed for a randomly chosen subset of 5% of the total number of reflections, which were not used during refinement. | |
| dRoot mean square deviation from ideal values. | |
