# Supplementary material for: Motor domain-mediated autoinhibition dictates axonal transport by the kinesin UNC-104/KIF1A
Source: PLoS Genet. 2021 Nov 29;17(11):e1009940. doi: 10.1371/journal.pgen.1009940 (PMC8659337; doi:10.1371/journal.pgen.1009940)
Supplement: S2 Table — (DOCX) [file pgen.1009940.s010.docx]

**S2 Table: Strains with genome-editing**

| Genotype | Starting worm | sgRNAs |
| --- | --- | --- |
| XD5358: *unc-104 (xd408 L170Q)* | N2 | sg-GTTCGTGAACATCCTTTACTTGG |
| XD5030: *unc-104(xd359 C184Y)* | *unc-104(L640F, C184Y)* | sg1-CAATCTTATATATCTGGTGGTGG  sg2-GGTGGTGGTTCTATTTGGGAAGG |
| XD5442: *unc-104 (xd475 L410F)* | *unc-104(L640F, L410F)* |  |
| XD5027: *unc-104 (xd366 G421E)* | *unc-104(L640F, G421E)* |  |
| XD5357: *unc-104 (xd407 K427A)* | N2 | sg1-TGGCTGAAATTGGAAAGACTTGG  sg2-AGAGGAAGAACTTCGTGATATGG |
| XD6444: *unc-104 (xd496 L428A)* | N2 |  |
| XD6451: *unc-104 (xd53 lfL640F, xd408 L170Q)* | *unc-104 (L170Q)* | sg1-CAATCTTATATATCTGGTGGTGG  sg2-GGTGGTGGTTCTATTTGGGAAGG |
| XD6454: *unc-104 (xd53 lfL640F, xd407 K427A)* | *unc-104 (K427A)* |  |
| XD5343: *unc-104(xd130 lfG1092E, xd359 C184Y)* | *unc-104(C184Y)* | sg1-CCAACAATAGTTGATCATGCTCA  sg2-GCTGGACCTGAATGGGCTGGCGG |
| XD5477: *unc-104 (xd130 lfG1092E, xd475 L410F)* | *unc-104 (L410F)* |  |
| XD5478: *unc-104 (xd130 lfG1092E, xd366 G421E)* | *unc-104 (G421E)* |  |
| XD6448: *unc-104(e1265 lfD1541N, xd359 C184Y)* | *unc-104(C184Y)* | sg1-CCGTGATGATCGAGATTTGGTTA  sg2-ATTCCGTGATGATCGAGATTTGG |
| XD6449*:unc-104 (e1265 lfD1541N, xd475 L410F)* | *unc-104 (L410F)* |  |
| XD6450:*unc-104 (e1265 lfD1541N, xd366 G421E)* | *unc-104 (G421E)* |  |
| XD5031: *xdKi3(UNC-104::GFP knock-in)* | N2 | sg1-CCATTGATGGCTGGACAAATGAA  sg2-GTACGCAATCAATCCATTGATGG |
| XD5338: *xdKi6* *[UNC-104(xd359 C184Y)::GFP knock-in]* | *unc-104(C184Y)* |  |
| XD6442: *xdKi60 [UNC-104(xd496 L428A)::GFP knock-in]* | UNC-104::GFP knock-in | sg1-TGGCTGAAATTGGAAAGACTTGG  sg2-AGAGGAAGAACTTCGTGATATGG |
| XD5336:*xdKi4[UNC-104(xd53 lfL640F)::GFP knock-in]* | *unc-104 (L640F)* | sg1-CGCAATCAATCCATTGATGG  sg2-GGACAAATGAAATTACATGG |
| XD6443: *xdKi61 [UNC-104(xd53 lfL640F, xd359 C184Y)::GFP knock-in]* | UNC-104(C184Y)::GFP knock-in | sg1-CAATCTTATATAAGCGGTGGTGG  sg2-AAGCGGTGGTGGCTCTATTTGGG |
